# Impact of the Level of Adherence to the DASH Diet on Blood Pressure: A Systematic Review and Meta-Analysis

**DOI:** 10.3390/metabo13080924

**Published:** 2023-08-07

**Authors:** Xenophon Theodoridis, Areti Triantafyllou, Lydia Chrysoula, Fotios Mermigkas, Violeta Chroni, Konstantina Dipla, Eugenia Gkaliagkousi, Michail Chourdakis

**Affiliations:** 1 Laboratory of Hygiene, Social and Preventive Medicine and Medical Statistics, School of Medicine, Faculty of Health Sciences, Aristotle University of Thessaloniki, 54124 Thessaloniki, Greece; xtheodoridis@auth.gr (X.T.); lchrysoula@auth.gr (L.C.); fotiosam@auth.gr (F.M.); violetac@auth.gr (V.C.); 2 3rd Clinic of Internal Medicine, Papageorgiou Hospital, School of Medicine, Faculty of Health Sciences, Aristotle University of Thessaloniki, 56403 Thessaloniki, Greece; artriant@auth.gr (A.T.); egkaliagkousi@auth.gr (E.G.); 3 Exercise Physiology & Biochemistry Laboratory, Department of Sport Sciences at Serres, Aristotle University of Thessaloniki, 62110 Thessaloniki, Greece; kdipla@phed-sr.auth.gr

**Keywords:** DASH diet, blood pressure, hypertension, synthesis

## Abstract

*Introduction*: the objective of our study was to systematically review the current literature and perform a meta-analysis to evaluate the effect of the level of adherence to the DASH diet on blood pressure. *Methods*: The identification of relevant studies, data extraction and critical appraisal of the included studies were performed independently by two reviewers. A random-effects model was employed to synthesize the available evidence using the standardized mean difference (SMD) as the appropriate effect size. *Results*: A total of 37 and 29 articles were included in the qualitative and quantitative analysis, respectively. The pooled effect for systolic blood pressure was SMD = −0.18 (95%CI: −0.32 to −0.04; I^2^ = 94%; PI: −0.93 to 0.57) and for diastolic blood pressure it was SMD = −0.13 (95%CI: −0.19 to −0.06; I^2^ = 94%; PI: −0.42 to 0.17). *Conclusions*: Our findings showed that greater adherence to the DASH diet has a beneficial effect on blood pressure compared to the lowest adherence. Increased compliance with DASH diet recommendations might also have a positive effect on cardiometabolic factors and overall health status. Future studies should aim to standardize the tools of adherence to the DASH diet and utilize rigorous study designs to establish a clearer understanding of the potential benefits of the level of adherence to the DASH diet in blood pressure management.

## 1. Introduction

Hypertension, defined as the consistently high pressure of blood flow within vessels, is the leading cause of cardiovascular events and all-cause mortality worldwide. Hypertension bears a correlation with the incidence of cardiovascular and renal detriment [1]. As of 2010, nearly one third of adults worldwide had hypertension. The increasing prevalence of hypertension is mainly attributed to the growing number of elderly people, the preference for unhealthy food options (diets rich in sodium and poor in potassium), smoking and the absence of exercise [2].

According to the current literature, the cornerstone of hypertension treatment includes anti-hypertensive drugs [3], as well as lifestyle alterations that consist of salt moderation, the restriction of alcohol and cigarettes, body weight diminution, exercise and dietary approaches [4]. Specifically, the Dietary Approaches to Stop Hypertension (DASH) diet, which comprises fruits, vegetables, fiber and low-fat dairy products in abundance, has been recommended as an efficient diet for regulating normal blood pressure measurements [5,6]. On the other hand, adherence to the DASH diet can be defined as the extent to which an individual may follow nutritional recommendations according to the DASH dietary pattern [7]. Accordingly, the DASH Score is calculated using information obtained from validated food frequency questionnaires in which low and high scores indicate poor and good adherence, respectively.

Several studies have demonstrated that the DASH diet holds a pivotal role in decreasing blood pressure, taking into consideration that people must be able and inclined to ensue this dietary pattern [5,8]. Therefore, proper adherence to the DASH diet is important in the prevention and treatment of elevated blood pressure measurements. Recently, a considerable number of observational studies have been conducted, regarding the effect of the DASH diet on cardiovascular events, including blood pressure measurements [9]. Nevertheless, the results of the available studies are contradictory. 

Thus, the aim of our study was to systematically review the current literature and perform a meta-analysis to evaluate the effect of the level of adherence to the DASH diet on blood pressure values.

## 2. Methods

### 2.1. Protocol

This systematic review and meta-analysis follow the Preferred Reporting Items for Systematic reviews and Meta-Analyses (PRISMA, 2020) [10] and Meta-Analysis of Observational Studies in Epidemiology Guidelines (MOOSE) (Supplementary Tables S1 and S2) [11]. The study protocol was registered in PROSPERO with ID CRD42022368688.

### 2.2. Search Strategy

The electronic databases PubMed, Scopus and Web of Science Core Collection were searched for the identification of eligible studies from inception to November 2022. We also searched the gray literature and the references of the included studies. Only studies published in the English language without a restriction on publication date were included. Keywords related to DASH diet and hypertension, such as “DASH diet”, “hypertension”, “blood pressure” and “diet” were used for PubMed and were modified accordingly for the remaining databases. The full search string can be found in Supplementary Table S3.

### 2.3. Eligibility Criteria

Observational and interventional studies, investigating the association between the level of adherence (high versus low) to the DASH diet and changes in blood pressure measurements in the adult population were included in our review. Studies that did not report any data for raw blood pressure measurements in adults were excluded. We also excluded studies involving the pregnant or pediatric population.

### 2.4. Outcomes

The primary outcome of our review was the difference in systolic and diastolic blood pressure measurements according to the adherence level to the DASH diet.

### 2.5. Data Extraction

Data from the included studies were extracted independently by two researchers (LC and VC) using an identical standardized data extraction form. Information regarding the study design, first author’s name, publication year, country, sample size, participant’s characteristics (age, sex, BMI, physical activity, smoking), comorbidities, use of anti-hypertensive medication, blood pressure measurements (systolic and diastolic) and the level of adherence to the DASH diet as reported by an assessment tool were abstracted for each study. With regard to the statistical data, we extracted the mean difference and standard deviations, as well as baseline and post-treatment values. In case of any missing data, authors were contacted for additional clarifications regarding data collection and accuracy. Any conflicts were resolved by consensus.

### 2.6. Quality Appraisal

The quality appraisal regarding the methodological validity of all included studies was evaluated by two independent researchers using the checklists developed by the Joanna Briggs Institute (JBI). Checklists were employed according to the study design of each included record (cohort, case–control and cross-sectional studies). The quality assessment was completed by answering the 11 questions of the JBI tool related to the study design, methodological validity and reliability. The risk of bias (RoB 2.0) tool and the Critical Appraisal Skills Programme (CASP) for the randomized controlled trial checklist were used to evaluate the quality of interventional studies. Any disagreement was resolved by a third reviewer.

### 2.7. Statistical Analysis

A meta-analysis was conducted for our outcome of interest. Blood pressure measurements were considered as a continuous variable. We used the mean, standard deviation and number of participants in each arm. When the included studies reported standard errors or 95% confidence intervals (95% CI) we transformed them to standard deviation following the guidelines by Cochrane. Furthermore, median values were transformed to mean values according to Wan and colleagues’ [12] approach. A random effects model was employed due to the expected heterogeneity between the included studies. Standardized mean differences (SMDs) and 95% confidence intervals (Cis) were used to present our findings. Heterogeneity was measured using tau-square (τ^2^) and the I^2^ index and estimated using the restricted maximum likelihood method. Funnel plots and Egger’s test were used for the evaluation of publication bias. We also performed subgroup and sensitivity analyses to explain heterogeneity and assess the robustness of our findings, respectively. All of the analyses were performed in the statistical software R Studio (version 2022.12.0 + 353) using the meta package.

### 2.8. Quality of the Evidence

The quality of our findings was assessed using the Grading of Recommendations Assessment, Development and Evaluation (GRADE), as recommended by the Cochrane handbook [13]. Domains such as the risk of bias, publication bias, heterogeneity, imprecision of the results and indirectness of the evidence were taken into consideration for the total evaluation. 

## 3. Results

### 3.1. Study Selection

**Figure 1:**
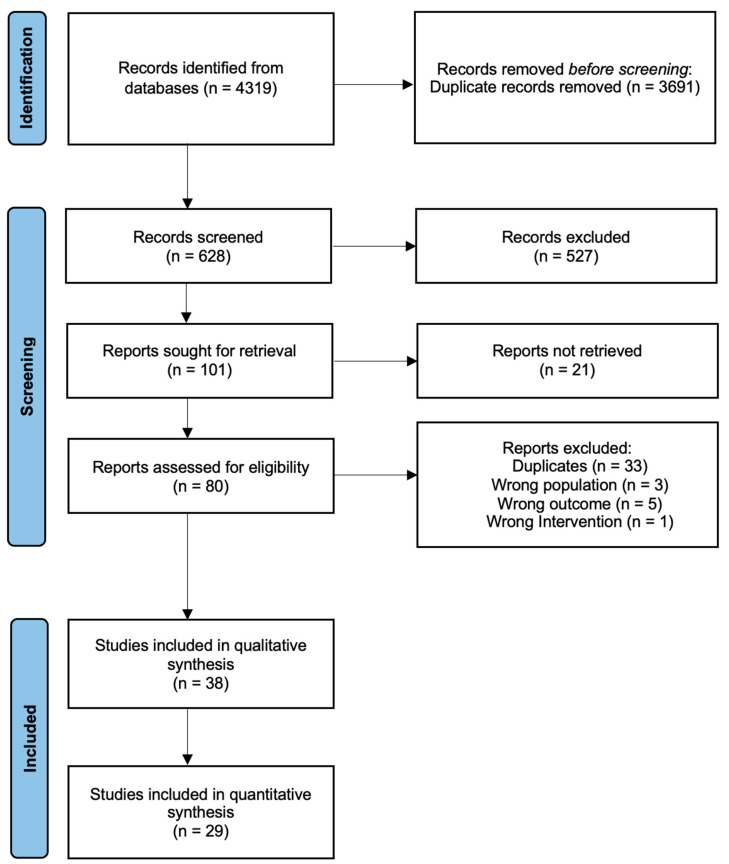
Flow diagram of the eligibility process.

Through the electronic database search, a total of 4319 records were identified, and after the removal of duplicates, 628 articles were reviewed for eligibility. Of those, 527 were excluded based on the title and abstract, 21 were removed due to a lack of access to the full-text articles and a total of 80 records remained for full-text assessment. In the qualitative synthesis, we included 37 papers, and of which, 3 were randomized trials, 20 were cohort studies, 1 was a case–control study and 14 were cross-sectional studies, while only 29 studies were included in the quantitative analysis (Figure 1).

### 3.2. Study and Patient Characteristics

The socio-demographic characteristics of the included studies are summarized in Table 1 [14,15,16,17,18,19,20,21,22,23,24,25,26,27,28,29,30,31,32,33,34,35,36,37,38,39,40,41,42,43,44,45,46,47,48,49,50]. Fourteen studies were conducted in the U.S. [14,20,21,27,29,30,36,38,39,41,43,48,49,50], two studies were conducted in the U.K. [33,42], Spain [26,45] and Italy [15,16], one study was conducted in Greece [18], Brazil [22], Ireland [28], Korea [34], the Netherlands [41], Sweden [46] and Turkey [37], six were conducted in Iran [25,31,32,35,44,47], three were conducted in China [19,23,24] and one study was conducted in four different countries [17]. The number of participants identified in the low- and high-adherence groups in each study ranged from 25 to 19,503 individuals. Adherence to the DASH diet was assessed using the tool constructed by Fung et al. (2008) [51] in 28 studies [15,16,17,18,19,20,23,24,26,27,28,29,30,31,32,33,35,36,38,40,41,42,43,45,46,47,49,50] and the tool developed by Mellen at al. (2008) [52] in three studies [14,39,48], while one study [25] implemented the tool by Valipour et al. (2017) [53], one study [34] implemented the tool by Lee et al. (2017) [54], one study [21] implemented the tool by Folsom et al. (2007) [55], one study [44] utilized principal component analysis (PCA) by Fransen et al., (2014) [56] and two studies [22,37] developed their DASH Score based on the guidelines produced by the National Institutes of Health and the National Heart Lung and Blood Institute (2018) [57]. Moreover, 18 studies [14,17,20,26,29,30,31,32,33,36,38,41,43,44,46,48,49,50] reported an intake of anti-hypertensive medication treatment by the participants including angiotensin-converting-enzyme inhibitors (ACEIs), angiotensin receptor blockers (ARBs) or any other anti-hypertensive agents.

Regarding patient characteristics, all details can be found in Table 2. In only one study [15] were participants disease-free, while in the remaining 35 studies [14,16,17,18,19,20,21,22,23,24,25,26,27,28,29,30,31,32,33,34,35,36,37,38,39,40,41,42,43,44,46,47,48,49,50], participants were diagnosed with depression, insomnia and cardiometabolic diseases including diabetes, obesity, hypertension, dyslipidemia, metabolic syndrome (MetS), chronic kidney disease (CKD), hyperuricemia, atrial fibrillation or diabetic nephropathy or had undergone surgery for leg amputation; for two [30,45] studies, relevant details were not provided. The mean BMI of all individuals ranged from 23.1 to 32.8 kg/m^2^, the mean SBP ranged from 102.5 to 154.1 mmHg and the DBP ranged from 45.8 to 88.8 mmHg.

### 3.3. SBP and DBP Levels

**Figure 2:**
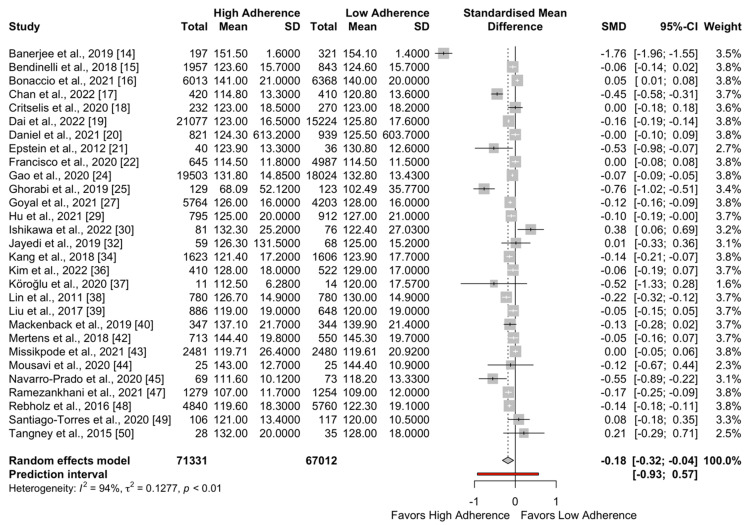
Meta-analysis for SBP (mmHg).

**Figure 3:**
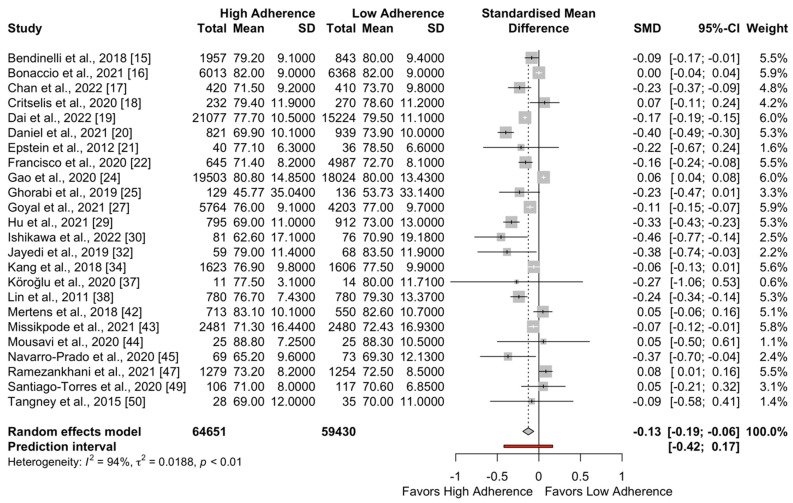
Meta-analysis results for DBP (mmHg).

The forest plots for SBP and DBP are presented in Figure 2 and Figure 3, respectively. The pooled effect for SBP favored the high adherence to the DASH diet compared to low adherence (SMD = −0.18; 95%CI −0.32 to −0.04; I^2^ = 94%; PI: −0.93 to 0.57). Regarding DBP, a significant difference was also observed favoring high adherence to the DASH diet (SMD = −0.13; 95%CI: −0.19 to −0.06; I^2^ = 94%; PI: −0.42 to 0.17).

### 3.4. Subgroup Analysis

There was a difference between the two groups regarding both SBP and DBP levels according to the use of drug prescription for hypertension. More specifically, high adherence to the DASH diet was associated with SBP values compared to low adherence for the participants that did not receive any anti-hypertensive medication (SMD = −0.14; 95%CI −0.22 to −0.06, I^2^ = 91%) (Supplementary Figure S1). Furthermore, a similar association was also observed for DBP values (SMD = −0.23; 95%CI −0.34 to −0.13, I^2^ = 84%) (Supplementary Figure S2). 

Furthermore, a subgroup analysis according to the study design of the included studies was conducted. There was no difference between the high and low adherence to the DASH diet on SBP when cohort or cross-sectional studies were pooled together. On the other hand, there was a significant difference favoring high adherence to the DASH diet based on the randomized controlled trial (Supplementary Figure S3). As far as DBP is concerned, a difference was observed when cohort or cross-sectional studies were synthesized. In contrast, a difference was absent in the randomized controlled trial (Supplementary Figure S4).

Lastly, we performed a subgroup analysis for subsets of studies such as different continents for the SBP and DBP outcomes. There was no difference in SBP between high and low adherence to the DASH diet when studies performed in North America, Europe and South America were synthesized. A significant difference was observed in one study, which was a multicenter one, and in the studies from Asia (Supplementary Figure S5). Regarding DBP, a difference between the two groups was present in the studies that were conducted in North and South America, as well as in the multicenter one (Supplementary Figure S6). 

### 3.5. Sensitivity Analysis

To explore high heterogeneity, we conducted a leave-one-out analysis for both of our outcomes. The findings of this sensitivity analysis showed that regarding SBP there was no significant change in heterogeneity values when omitting one study each time (Supplementary Figure S7). The same findings apply to the DBP outcome (Supplementary Figure S8). 

### 3.6. Risk of Bias Assessment

As depicted in Supplementary Tables S4–S6, almost all cohort and cross-sectional studies successfully performed the recruitment process of participants, identified the potential confounding factors, and used valid methods for measuring the exposures and outcomes. However, information on the sufficient follow-up time, the potential reasons regarding incomplete follow-up, and information on the implementation of strategies for addressing this matter were either missing or were not described clearly. With reference to the interventional studies (Supplementary Table S7), the overall quality was rated as having “some concerns”, according to the RoB 2.0 tool.

### 3.7. Publication Bias

According to the funnel plots, there were no signs of publication bias in our review (Supplementary Figures S9–S10). Moreover, Egger’s test for the SBP was *p* = 0.355 and for DBP it was *p* = 0.232, indicating the absence of publication bias.

### 3.8. Certainty of Findings

Based on the GRADE approach, the certainty of our evidence was judged as being very low for both of our outcomes of interest.

## 4. Discussion

The present systematic review and meta-analysis aimed to evaluate the impact of the level of adherence to the DASH diet on blood pressure based on synthesizing the available data from observational and interventional studies. Our findings demonstrate a difference in the reported values of SBP and DBP between participants in the highest and lowest adherence group.

The results of our review support the notion that higher adherence to the DASH diet may have a favorable effect on SBP. However, they should be interpreted with caution due to the high heterogeneity among the included studies. This beneficial effect of the DASH diet could be attributed to its dietary characteristics and the combination of various foods including the high consumption of fruits, vegetables, whole grains, and nuts and the limited salt intake, which have been associated with numerous studies with a reduction in blood pressure [58].

With regard to SBP, high adherence to the DASH diet had a beneficial effect compared to low adherence. It should be stated that few of the included studies presented a mean SBP > 140 mmHg, while in parallel, the majority of them presented a mean DBP < 130 mmHg. This finding is essential, as it supports the protective role of high adherence to the DASH diet in SBP even in subjects with normal SBP.

Regarding DBP, the level of adherence to the DASH diet led to a difference between the highest and lowest adherence group. It should be noted that none of the included studies presented a mean DBP > 90 mmHg, while in parallel, the majority of them presented a mean DBP < 80 mmHg. This finding is of great importance, as it supports that high adherence to the DASH diet could reduce DBP values even in subjects with normal DBP.

In line with our results, published systematic reviews and meta-analyses investigating the effectiveness of the DASH diet provided as an intervention, compared to the usual diet group, showed that the DASH diet is effective in reducing both systolic and diastolic blood pressure [58,59,60]. Furthermore, the DASH diet is also effective in lessening other cardiovascular risk factors such as the concentrations of total and LDL cholesterols. HbA1c and insulin concentrations as well as body weight were also reduced in participants assigned to the dietary intervention group compared to the control group, as demonstrated by an umbrella review of systematic reviews and meta-analyses [61].

It should be noted that the DASH diet given exclusively as a dietary intervention to individuals might promote different health outcomes compared to those that emerged from simply measuring adherence to the DASH diet with the use of specific tools. It is possible for dietary interventions to not enhance compliance with a particular dietary pattern as they also require participants’ adherence. On the contrary, dietary adherence demonstrates the degree of compliance to a diet that is directly related to individuals’ preferences, without corresponding to the consumption of a specified dietary plan. In addition, the level of diet adherence may be affected by various factors, including socioeconomic status, medical history, self-efficacy, level of education, religion, and place of residence, as well as psychological factors and individuals’ attitudes [62].

The DASH diet is not only effective in reducing cardiometabolic outcomes, but there are also published syntheses demonstrating that higher adherence to the DASH diet has a protective role in developing type 2 diabetes mellitus [63] and cardiovascular diseases (CVDs) [64] such as coronary heart disease and stroke [65], and also leads to a significant reduction in all-cause, cancer, and CVD mortality [64]. Lastly, a recently published protocol (PROSPERO 2022 CRD42022344686) of a systematic review and meta-analysis aimed to evaluate adherence to the DASH diet and hypertension risk [66]. The authors found that higher adherence to the DASH diet was associated with a reduced risk of hypertension incidence compared to the lowest adherence to the DASH diet.

To the best of our knowledge, this is the first systematic review and meta-analysis that has investigated the association between adherence to the DASH diet and blood pressure levels. It is also worth noting that our study had certain limitations. Firstly, the study design of the majority of the included studies, i.e., observational studies, limits the confidence in our findings. Furthermore, we are unable to establish causality between adherence to the DASH diet and blood pressure outcomes using observational studies. Secondly, the high heterogeneity observed among the included studies could affect the reliability of the findings; hence, they should be cautiously interpreted. Lastly, we used data from crude models as our outcome of interest was not reported in adjusted analyses.

In conclusion, our findings showed that greater adherence to the DASH diet has a significant effect on blood pressure levels compared to the lowest adherence. Increased compliance with DASH diet recommendations might also have a positive effect on cardiometabolic factors and overall health status. Future studies should aim to standardize the tools of adherence to the DASH diet and utilize rigorous study designs to establish a clearer understanding of the potential benefits of the level of adherence to the DASH diet in blood pressure management and monitoring.

## Figures and Tables

**Table 1:** Study characteristics included in the systematic review.

Study ID	Country	Study Design	Population	No. of Participants (Low/High)	Mean Age (SD)	Exclusion Criteria	DASH Assessment Tool	Use of Anti-Hypertensive Medication
Benerjee et al., 2019 [14]	U.S.	Prospective observational study	Adults with hypertension and CKD enrolled in the National Health and Nutrition Examination Survey (NHANES) III	321/197 Total: 1110	70.2 ± 12.9	Missing data on dietary intake, eGFR < 30 or >59 mL/min, pregnancy	DASH Score by Mellen et al. (2008)	ACEI, ARB
Bendinellii et al., 2019 [15]	Italy	Cross-sectional observational study	Residents of Florence and Prato	843/1959 Total: 10,163	50.4 ± 7.7	Diagnosis of hypertension or anti-hypertensive drugs at any time in the past	DASH Score by Fung et al. (2008)	No
Bonaccio et al., 2020 [16]	Italy	Prospective observational study	Men and women from the general population of Moli-sami Study	6368/6013 Total: 12,381	55.0 ± 12.0	EI < 800 kcal/day in men and <500 kcal/day in women or >4000 kcal/day in men and >3500 kcal/day in women, unreliable medical dietary questionnaires, lost to follow-up, missing data on outcome exposure, missing information on the main covariates of interest	DASH Score by Fung et al. (2008)	No
Chan et al., 2022 [17]	China, Japan, U.K., U.S.	Cross-sectional observational study	Adults	410/420 Total: 2164	28.9 ± 5.9	Incomplete dietary data	DASH Score by Fung et al. (2008)	Yes
Critselis et al., 2019 [18]	Greece	Prospective observational study	Greek male adults, free of CVD	965/1054 Total: 2019	45.2 ± 14.0	CVD at baseline	DASH Score by Fung et al. (2008)	No
Dai et al., 2022 [19]	China	Prospective observational study	Adults from Tibetan, Yi, Miao, Bai, Bouyei and Dong ethnic groups	No info Total: 81,433	50.5 ± 11.2	<30 y or >79 y, missing information on diet-related variables, missing information on outcome-related data, implausible BMI values (BMI < 14 or >45 kg/m^2^), unusual daily EI (<600 or >3500 kcal/d for females, <800 or >4200 kcal/d for males), self-reported physician-diagnosed hypertension and use of anti-hypertensive medication	DASH Score by Fung et al. (2008)	No
Daniel et al., 2021 [20]	U.S.	Prospective observational study	Chinese, Hispanic, non-Hispanic Black or non-Hispanic white	4169 Total: 1760	60.4 ± 9.5	Extreme EI of < 500 kcal or >5000 kcal, without FFQ, no cognitive data, using Alzheimer’s medications	DASH Score by Fung et al. (2008)	Yes
Epstein et al., 2012 [21]	U.S.	RCT	Healthy, overweight or obese men and women with above-normal BP	40/26 Total: 144	51.3 ± 9.0	Medication, other comorbidities, too high/low BMI and BP, dietary reasons	DASH Score by Folsom et al. (2007)	No
Fransisco et al., 2020 [22]	Brazil	Prospective observational study	Active or retired civil servants of higher education and research institutions	4987/645 Total: 5632	49.9 ± 8.3	Fulfilled the criteria for hypertension, anti-hypertensive drugs, reported CVD, missing information on BP values, dietary reasons, urinary Na, race/skin color	DASH Score developed based on guidelines by the National Institutes of Health and National Heart Lung and Blood Institute (2018)	No
Gao et al., 2021 [23]	China	RCT	Chinese adults with hyperilipidemia	No info Total: 269	58.0 ± 8.0	Known chronic diseases, acute and chronic infectious diseases, trauma or surgery, use of hormonal therapies, use of medications known to influence lipid metabolism within the past six months, use of anti-inflammatory or antibiotic drugs within the past three months, use of vasomotor function drugs within the past three months, taking phytochemicals or other dietary supplements within the past two months and pregnant or lactating women	DASH Score by Fung et al. (2008)	No
Gao et al., 2020 [24]	China	Prospective observational study	Adults from Tangshan City	18,024/19,503 Total: 71,893	51.4 ± 0.1	Previously diagnosed as having gout, reported an implausible EI (<800 kcal/day or >4000 kcal/day for men, and <500 kcal/day or >3500 kcal/day for women), poor results on food frequency questionnaires, incomplete information on demographic data	DASH Score by Fung et al. (2008)	No
Ghorabi et al., 2019 [25]	Iran	Cross-sectional observational study	Iranian adults	136/129 Total: 396	38.2 ± 9.5	Pregnancy, post-menopausal status, lactation, any kind of cancers, medication for modifying fat, blood sugar and BP, ischemic heart disease, use of sedative or hypnotic drug, use of anti-histamine, use of immune system inhibitors, following any special diet for any reasons under the supervision of a diet therapist, being a professional athlete, use of weight loss drug	DASH Score by Valipour et al. (2017)	No
Glenn et al., 2021 [26]	Spain	RCT	Older men and women with BMI 27–40 kg/m^2^ and fulfilled at least three criteria of the MetS	2026/1636 Total: 6874	65.0	Implausible EI (<500 or >3500 kcal/d for women and <800 or >4000 kcal/d for men) or missing information on FFQ at baseline	DASH Score by Fung et al. (2008)	Yes
Goyal et al., 2021 [27]	U.S.	Prospective observational study	African-American and white adults	4203/5764 Total: 18,856	64.0 ± 9.2	Missing or incomplete FFQ (≤85%), implausible EI (men <3347 kJ/d or >20,920 kJ/d, and women <2093 kJ/d or >18,841)	DASH Score by Fung et al. (2008)	No
Harrington et al., 2013 [28]	Ireland	Cross-sectional observational study	Men and women based in a primary care setting in the North Cork Region of the Republic of Ireland	No info Total: 2047	60.7	Duplicates, deaths and ineligibles, mortality, lost to follow-up, too unwell to participate	DASH Score by Fung et al. (2008)	No
Hu et al., 2021 [29]	U.S.	Prospective observational study	Men and women with an estimated eGFR 20–70 mL/min/1.73 m^2^	912/795 Total: 2403	57.3 ± 11.3	Unfilled FFQ, extreme self-reported EI (women: <500 or >3500 kcal/d; men: <700 or >4500 kcal/d), not sufficient data to calculate all dietary pattern scores, missing covariates of interest	DASH Score by Fung et al. (2008)	ACEI, ARB
Ishikawa et al., 2022 [30]	U.S.	Cross-sectional observational study	Adults with self-reported diagnosis of HF	76/81 Total: 348	65.3 ± 0.9	Did not attend the mobile examination center morning session, incomplete data on fasting plasma glucose and insulin to calculate the HOMA-IR, physician diagnosis of DM or used diabetes medications, pregnancy, implausible EI (gender-specific <1st and >99th percentiles of EI per day)	DASH Score by Fung et al. (2008)	ACEIs, ARBs, beta-blockers, loop diuretics
Jalilpiran et al., 2020 [31]	Iran	Cross-sectional observational study	Older adult men living in southern Tehran	203/154 Total: 357	64.9 ± 6.5	Malignant diseases (e.g., cancer), under- or over-reported total EI (<800 kcal/day and >4200 kcal/day), under- and over-reporting of total EI	DASH Score by Fung et al. (2008)	No info
Jayedi et al., 2019 [32]	Iran	Case–control study	Women with type 2 DM and diabetic nephropathy at Kowsar Diabetes Clinic in Semnan	No info Total: 210	55.3 ± 7.0	GDM, type 1 DM, medication treatment, previous history of cancer, myocardial infarction, hepatic disease, autoimmune disorders, stroke and coronary angiography	DASH Score by Fung et al. (2008)	beta-blockers, ACEIs, ARBs
Jones et al., 2018 [33]	U.K.	Prospective observational study	Men and women participating in general practices in Norfolk	5744/4181 Total: 23,655	59.1	Missing FFQ data, missing baseline CVD data, missing covariate data, incorrect date of death	DASH Score by Fung et al. (2008)	Yes
Kang et al., 2018 [34]	Korea	Cross-sectional observational study	Post-menopausal women from South Korean population	1606/1623 Total: 6826	58.5 ± 6.3	Missing clinical data, DM, extremely low or high EI (<500 kcal or 5000 kcal)	DASH Score by Lee et al. (2017)	No
Khodarahmi et al., 2021 [35]	Iran	Cross-sectional observational study	Healthy obese adults in the city of Tabriz	No info Total: 347	38.0 ± 7.4	Pregnancy, lactation, menopausal women, medical history of chronic diseases (CVD, hypertension, hyperlipidemia, DM, renal diseases, hepatic disorders and cancer), recent surgery such as bariatric surgery, any medications and supplements which had effects on weight and variables studied such as loop diuretics, corticosteroids, antidepressants, statins and anti-hypertensive agents, EI outside of the range of 800–4200 kcal/day	DASH Score by Fung et al. (2008)	No
Kim et al., 2022 [36]	U.S.	Prosopective observational study	Men and women of African American, Hispanic, Asian, Indian, Pacific Islander and Native American origins	522/410 Total: 1899	67.0 ± 9.0	Missing information on diets and covariates, missing mortality	DASH Score by Fung et al. (2008)	Yes
Köroğlu et al., 2020 [37]	Turkey	Cross-sectional observational study	Male patients with at least one year and maximum three years of amputation history	No info Total: 35	36.9 ± 9.3	DM, hypertension, thyroid dysfunction, amputees due to vascular problems	DASH Score developed based on guidelines by the National Institutes of Health and National Heart Lung and Blood Institute (2018)	No
Lin et al., 2011 [38]	U.S.	Prospective observational study	U.S. female nurses	780/780 Total: 3121	67.0	No cumulative average dietary pattern data available, no measured plasma creatine in sample collection	DASH Score by Fung et al. (2008)	ACEI, ARB
Liu et al., 2017 [39]	U.S.	Prospective observational study	African American and white people from U.S. census tracts in Baltimore City, Maryland	648/886 Total: 1534	48.0	Did not undergo serum creatinine at baseline, no dietary intake data, eGFR <60 mL/min per 1.73 m^2^ at baseline, survived but did not undergo a follow-up serum creatinine measurement	DASH Score by Mellen et al. (2008)	No
Mackenbach et al., 2019 [40]	The Netherlands	Cross-sectional observational study	Adults (Netherlands Study of Depression and Anxiety)	344/347 Total: 1543	52.4 ± 12.9	Incomplete FFQ, extreme EI, missing data on their six-digit postcode, hypertensive medication	DASH Score by Fung et al. (2008)	No
Mattei et al., 2017 [41]	U.S.	Propsective observational study	Self-identified Puerto Ricans residing in Boston	No info Total: 1189	Low: 55.3 ± 7.1 High: 58.8 ± 7.3	Unable to answer questions due to serious health conditions, planned to move away from the area within two years, low MMSE score (≤10)	DASH Score by Fung et al. (2008)	Yes
Mertens et al., 2017 [42]	U.K.	Prospective observational study	Middle-aged men from the town of Caerphilly and adjoining villages, South Wales (U.K.)	550/713 Total: 1867	56.6 ± 4.3	Men who died, history of myocardial infarction or stroke, DM, missing dietary data	DASH Score by Fung et al. (2008)	No
Missikpode et al., 2021 [43]	U.S.	Prospective observational study	Adults self-identified as Hispanic/Latino	2480/2481 Total: 9921	41.0 ± 0.28	Missing information on kidney-function measures, incomplete diet data, missing data on covariates, CKD at baseline	DASH Score by Fung et al. (2008)	ACEI, ARB
Mousavi et al., 2020 [44]	Iran	Cross-sectional observational study	Adults with mild to moderate hypertension	25/25 Total: 101	40.7 ± 4.48	Angina pectoris, type 1 DM, renal diseases, pregnancy and lactation, special diet and intake of supplements	DASH Score derived from PCA (Fransen et al., 2014)	Yes
Navarro-Prado et al., 2020 [45]	Spain	Cross-sectional observational study	University students during the 2013–2014 academic year	73/69 Total: 244	22.4 ± 4.76	Accepted and signed an informed consent document, previously diagnosed with an endocrine disease, lacking anthropometric, dietary or demographic data, ≥32 years old	DASH Score by Fung et al. (2008)	No
Nilsson et al., 2019 [46]	Sweden	Cross-sectional observational study	Community-dwelling women	No info Total: 112	67.0 ± 1.6	CHD and DM, disability with respect to mobility, using prescribed anti-inflammatory medication, smokers, incomplete data on PA, incomplete data on inflammatory and metabolic biomarkers	DASH Score by Fung et al. (2008)	Yes
Ramezankhani et al., [47]	Iran	Prospective observational study	Adult residents of Tehran participating in Tehran Lipid and Glucose Study (TLGS)	1254/1279 Total: 4793	38.9 ± 12.7	Under- or over-reporters of EI (<800 or ≥4200 kcal/day), hypertension at baseline, missing data on hypertension status without any follow-up data	DASH Score by Fung et al. (2008)	No
Rebholz et al., 2016 [48]	U.S.	Prospective observational study	Participants of Atherosclerosis Risk in Communities Study (ARIC), predominantly African American and white with baseline eGFR ≥60 mL/min/1.73 m	5759/4840 Total: 14,882	54.1 ± 5.7	Missing dietary ΕΙ data, implausibly low caloric intake (<600 kcal for men and <500 kcal for women) and implausibly high caloric EI (>4200 kcal for men and >3600 kcal for women), baseline eGFR <60 mL/min/1.73 m^2^ or ESRD, identified by linkage to the US Renal Data System registry, neither African American nor white, missing covariates	DASH Score by Mellen et al. (2008)	ACEI, ARB
Santiago-Torres et al., 2020 [49]	U.S.	Prospective observational study	Post-menopausal women of Mexican ethnic descent who participated in the Women’s Health Initiative (WHI)	117/106 Total: 334	58.6 ± 6.4	Non-Mexican, American or Chicana, metabolic syndrome, diabetes, participated in the intervention group for the Dietary Modification trial, either low or high self-reported EI from the FFQ (<500 or >4000 kcal)	DASH Score by Fung et al. (2008)	Yes
Tangney et al., 2015 [50]	U.S.	Cross sectional observational study	Older Latino adults from CAPACES (who had a score less than 14 on a 21-point Mini-Mental State Examination)	Fung: 35/28	66.0 ± 9.0	Less than 50 years old, score <14 on the shortened MMSE, too young, used a walking assistive device, not Latino	DASH Score by Fung et al. (2008)	Yes

ACEI: angiotensin-converting-enzyme inhibitor; ARB: angiotensin receptor blockers; BMI: body mass index; BP: blood pressure; CHD: coronary heart disease; CKD: chronic kidney disease; CVD: cardiovascular disease; DASH: dietary approaches to stop hypertension; DM: diabetes mellitus; eGFR: estimated glomerular filtration rate; EI: energy intake; ESRD: end-stage renal disease; HOMA-IR: homeostatic model assessment of insulin resistance; FFQ: Food Frequency Question-naire; GDM: gestational diabetes mellitus; HF: heart failure; MetS: metabolic syndrome; MMSE: mini-mental state examination; PCA: principal component analysis; RCT: randomized controlled trial.

**Table 2:** Patients’ health characteristics of the included studies.

Study ID	Comorbidities (Low/High) *	Percentage (%) of Participants with HTN (Low/High)	BMI (Low/High) *	SBP (Low/High) *	DBP (Low/High) *	Physical Activity (Low/High) *	Smoking Status (Low/High)	Sodium Intake (mg)	Potassium Intake (mg)
Benerjee et al., 2019 [14]	CKD	No info	26.5 ± 4.9/ 28.7 ± 6.0	154.1 ± 1.4/ 151.5 ± 1.6	No info	Moderate: 96.0%/93.8% Intense: 4.0%/6.2%	Current: 22.0%/6.3% Past: 37.4%/53.4 Never: 40.6%/40.3%	1809.9 ± 26.0/ 1597.9 ± 48.1	1227.7 ± 15.1/ 2249.6 ± 35.4
Bendinellii et al., 2019 [15]	No	No info	Under/normal weight: 55.9%/53.9% Overweight: 33.9%/35.6% Obesity: 10.2%/10.6%	124.6 ± 15.7/ 123.6 ± 15.7	80.0 ± 9.4/ 79.2 ± 9.1	Inactive: 22.5%/16.6% Moderately inactive: 23.4%/23.9% Moderately active: 45.0%/47.7% Active: 9.1%/11.7%	Current: 34.2%/24.5% Former: 25.5%/31.3% Never smoked: 40.3%/44.3%	2740.0 ± 9.9/2640.0 ± 11.3	No info
Bonaccio et al., 2020 [16]	Obesity, DM (3.7%/5.6%), Hyperlipidemia (5.3%/10.3%)	22.5%/31.5%	Obesity: 29.1%/29.3%	140.0 ± 20.0/ 140.0 ± 21.0	82.0 ± 9.0/82.0 ± 9.0	Leisure-time PA (MET-h/day): 42.6%/56.5%	Current: 27.2%/19.4%	No info	No info
Chan et al., 2022 [17]	CVD (42.2%/33.8%)	No info	30.5 ± 6.3/ 26.9 ± 5.0	120.8 ± 13.6/ 114.8 ± 13.3	73.7 ± 9.8/ 71.5 ± 9.2	Μoderate or heavy (hours/day): 4.0 ± 3.7/3.0 ± 2.9	Current: 31.2%/5.5%	No info	No info
Critselis et al., 2019 [18]	Hypercholesterolemia (40.6%/44.5%), DM (7.4%/7.0%), MetS (18.4%/20.9%)	29.9%/33.0%	26.1 ± 4.4/ 26.5 ± 4.6	123.0 ± 18.2/ 123.0 ± 18.5	78.6 ± 11.2/ 79.4 ± 11.9	38.9%/42.7%	42.5%/42.6%	No info	No info
Dai et al., 2022 [19]	Hypertension, Depression, Insomnia	25.7%/20.4%	24.2 ± 3.6/ 23.9 ± 3.3	125.8 ± 17.6/ 123.0 ± 16.5	79.5 ± 11.1/ 77.7 ± 10.5	29.1 ± 19.7/ 24.4 ± 16.7 (MET hours/day)	Never: 74.7%/76.6% Previous: 21.8%/17.7% Current: 3.5%/5.7%	No info	No info
Daniel et al., 2021 [20]	DM (9.3%/7.5%)	40.3%/41.4%	29.3 ± 5.3/ 27.4 ± 5.0	124.3 ± 19.7/ 125.5 ± 21.4	73.9 ± 10.0/ 69.9 ± 10.1	1456.1 ± 2631.8/ 1956.6 ± 2641.9 (MET min/week)	Current: 21.2%/4.9%	No info	No info
Epstein et al., 2012 [21]	Obesity	Total: 47%	No info	129.2 ± 1.9/ 134.5 ± 2.2	76.6+1.1/ 80.9+1.3	No info	No info	No info	No info
Fransisco et al., 2020 [22]	DM (8.1%/9.5%)	No info	25.8 ± 4.2/ 24.9 ± 3.8	114.5 ± 11.5/ 114.5 ± 11.8	72.7 ± 8.1/ 71.4 ± 8.2	Light: 78.6%/62.8% Μoderate: 14.1%/24.9% Vigorous: 7.3%/12.4%	Non-smoker: 58.8%/65.3% Former: 25.8%/25.4% Smokers: 15.4%/9.3%	No info	3982.0 ± 1607.0/ 5260.0 ± 1664.0
Gao et al., 2021 [23]	Central obesity (total 44.2%)	No info	Underweight (total 12%) Overweight (total 39.4%) Obesity (total 11.5%)	No info	No info	No info	No: 93.3% Yes: 6.7%	No info	No info
Gao et al., 2020 [24]	CHD (1.6%/2.5%) Hyperuricemia (18.3%/14.4%)	No info	24.7 ± 0.03/ 24.8 ± 0.03	132.8 ± 0.1/ 131.8 ± 0.1	80.0 ± 0.1/ 80.8 ± 0.1	Low: 29.4%/48.1% Μoderate: 21.1%/8.6% High: 33.0%/26.0% Unknown: 16.4%/17.2%	No: 51.5%/58.5% Yes: 48.5%/41.5%	No info	No info
Ghorabi et al., 2019 [25]	Components of MetS: Abdominal obesity: 30.6%/36.1%, Elevated BP: 47.3%/22.1%, High TG: 43.5%/23.2%, Reduced HDL: 27.7%/40.1%, Abnormal GL: 41.0%/32.7%	No info	28.7 ± 4.9/ 28.5 ± 4.9	102.5 ± 35.8/ 68.1 ± 52.1	53.7 ± 33.1/ 45.8 ± 35.0	No info	Current: 35.3%/23.5%	No info	No info
Glenn et al., 2021 [26]	DM (29.0%/32.0%), Hypercholesterolemia (76.0%/75.0%)	93.0%/94.0%	32.8 ± 3.5/ 32.1 ± 3.4	No info	No info	2193.0 ± 2154.0/ 2856.0 ± 2444.0 (MET min/week)	Never: 41.0%/48.0% Former: 44.0%/42.0% Current: 15.0%/32.1%	No info	No info
Goyal et al., 2021 [27]	Atrial fibrilation (7.3%/7.3%), DM (14.9%/17.3%)	57.4%/53.8%	29.0 ± 6.2/ 28.0 ± 5.7	128.0 ± 16.0/126.0 ± 16.0	77.0 ± 9.7/76.0 ± 9.1	4 or more times/week: 24.4%/32.0% 1 to 3 times/week: 34.7%/39.9% None: 40.9%/28.2%	Current: 26.0%/9.5% Past: 36.9%/42.1% Never: 37.2%/48.4%	No info	No info
Harrington et al., 2013 [28]	Hypertension	33.6%/27.3%	No info	131.3 ± 16.4/ 126.8 ± 16.6	80.9 ± 9.9/ 79.8 ± 9.6	No info	No info	No info	No info
Hu et al., 2021 [29]	CKD, DM (37.0%/49.0%)	85.0%/79.0%	32.0 ± 8.0/ 32.0 ± 8.0	127.0 ± 21.0/ 125.0 ± 20.0	73.0 ± 13.0/ 69.0 ± 11.0	204.0 ± 135.0/ 198.0 ± 118.0 (METs/week)	21.0%/5.0%	2922.0 ± 1415.0/ 2788.0 ± 1268.0	2723.0 ± 1240.0/ 3311.0 ± 1313.0
Ishikawa et al., 2022 [30]	No info	No info	No info	122.4 ± 3.1/ 132.3 ± 2.8	70.9 ± 2.2/ 62.6 ± 1.9	No info	51.3%/4.3%	No info	No info
Jalilpiran et al., 2020 [31]	Any disease (dyslipidemia, HTN, abnormal GL levels) 60.9%/39.1%	No info	25.7 ± 2.8/ 25.3 ± 3.4	No info	No info	No info	51.0%/14.8%	No info	3710.0 ± 62.5/ 4528.8 ± 71.6
Jayedi et al., 2019 [32]	Type 2 DM, Diabetic nephropathy	No info	27.5 ± 4.6/ 28.7 ± 3.8	125.0 ± 15.2/ 126.3 ± 13.15	83.5 ± 11.9/ 79.0 ± 11.4	Low: 28.4%/32.9% Moderate: 35.8%/36.7% High: 35.8%/30.4%	No info	No info	No info
Jones et al., 2018 [33]	DM (4.1%/4.1%)	No info	No info	136.8/135.0	83.4/81.5	Inactive: 1953/920 Active: 3791/3261	Current: 19.0%/6.0%	No info	No info
Kang et al., 2018 [34]	MetS	No info	24.3 ± 3.1/ 24.0 ± 2.9	123.9 ± 17.7/ 121.4 ± 17.2	77.5 ± 9.9/ 76.9 ± 9.8	47.0%/54.7%	Non-smoker: 92.3%/94.9% Ex-smoker: 1.6%/2.0% Current smoker: 6.1%/3.1%	No info	No info
Khodarahmi et al., 2021 [35]	Obesity, Depression, MetS	No info	No info	120** (105.0, 130.0)/110.0** (110.0, 130.0)	77.5 ± 12.6/70.4 ± 16.6	*Men* Low: 35.3%/35.3% Moderate: 46.9%/12.5% High: 26.7%/23.3% *Women* Low: 33.9%/25.0% Moderate: 45.0%/30.0% High: 31.3%/18.8%	No info	No info	No info
Kim et al., 2022 [36]	Type 2 DM (63.0%/46.0%)	No info	29.0 ± 6.0/ 27.0 ± 5.0	129.0 ± 17.0/ 128.0 ± 18.0	No info	Score ^1^: 35.0 ± 6.0/ 36.0 ± 5.0	Current: 14.0%/14.0% Former: 36.0%/32.0%	No info	No info
Köroğlu et al., 2020 [37]	Traumatic lower limb amputation	No info	31.0 ± 7.7/ 24.1 ± 2.5	120.0 ± 17.6/112.5 ± 6.3	80.0 ± 11.7/ 77.5 ± 3.1	No info	No info	No info	No info
Lin et al., 2011 [38]	DM (24.6%/20.3%), hypercholesterolemia (65.0%/66.4%), CVD (6.8%/5.3%)	56.5%/48.3%	27.3 ± 1.3/ 25.1 ± 0.9	130.0 ± 3.2/ 125.0 ± 3.2	79.5 ± 2.9/ 77.5 ± 1.6	8.8 ± 2.5/ 18.9 ± 3.9 (METs/week)	Current: 11.6%/2.2% Ever: 56.3%/48.4%	2007.5 ± 67.5/ 1923.5 ± 60.4	No info
Liu et al., 2017 [39]	Obesity (42.4%/41.1%), DM (15.5%/15.6%)	42.1%/43.0%	29.7 ± 7.6/ 29.8 ± 7.8	120.0 ± 19.0/ 119.0 ± 19.0	No info	No info	Current: 52.3%/41.7% Former: 20.0%/21.4% None: 27.8%/36.9%	No info	No info
Mackenbach et al., 2019 [40]	Depression	17.6%/12.6%	26.7 ± 4.8/ 25.2 ± 4.0	139.9 ± 21.4/ 137.1 ± 21.7	No info	No info	Current: 38.4%/13.0%	No info	No info
Mattei et al., 2017 [41]	DM (36.4%/37.4%), CVD (19.4%/25.9%), Obesity (53.4%/57.4%)	68.2%/70.4%	31.8 ± 6.9/ 31.7 ± 6.3	135.0 ± 21.0/ 136.0 ± 19.0	82.1 ± 11.9/ 79.4 ± 9.8	Score ^2^: 31.0 ± 3.8/ 32.0 ± 4.6	Current: 31.1%/13.8%	No info	No info
Mertens et al., 2017 [42]	CVD	No info	25.5 ± 3.5/ 27.1 ± 3.4	145.3 ± 19.7/ 144.4 ± 19.8	82.6 ± 10.7/ 83.1 ± 10.1	Active: 42.4%/44.3%	Current: 61.8%/28.9%	2575.0 ± 596.7/ 2134.8 ± 577.3	No info
Missikpode et al., 2021 [43]	DM (12.0%/19.0%), CVD (21.0%/25.0%)	21.0%/24.0%	29.6 ± 9.5/ 29.4 ± 8.9	119.6 ± 20.9/119.7 ± 26.4	72.4 ± 16.9/71.3 ± 16.4	Low PA level: 44.0%/42.0%	Current: 28.0%/12.0%	No info	No info
Mousavi et al., 2020 [44]	Mild to moderate HTN	No info	29.7 ± 4.3/ 29.1 ± 5.1	144.4 ± 10.9/ 143.0 ± 12.7	88.3 ± 10.5/ 88.8 ± 7.25	4192.5 ± 6088.1/ 4132.3 ± 5508.6 (MET/min/week)	No info	3338.7 ± 978.7/ 2949.2 ± 320.2	2011.9 ± 694.5/ 2030.4 ± 915.6
Navarro-Prado et al., 2020 [45]	No info	No info	23.1 ± 4.1/ 23.1 ± 3.89	118.2 ± 13.3/ 111.6 ± 10.1	69.3 ± 12.1/65.2 ± 9.6	PAQ-C summary score: 3.9 ± 0.8/ 4.1 ± 0.8	No info	2800.0 ± 940.0/2400.0 ± 1130.0	2400.0 ± 850.0/ 2600.0 ± 1140.0
Nilsson et al., 2019 [46]	Obesity, Dyslipidemia	No info	No info	134.0 ± 15.0/ 139.0 ± 14.0	79.0 ± 9.0/ 79.0 ± 7.0	Daily time in moderate to vigorous PA (min): 23.0 ± 16.0/ 30.0 ± 24.0	No info	No info	No info
Ramezankhani et al., [47]	DM (3.3%/7.7%)	No info	26.0 ± 4.6/ 27.5 ± 4.5	109.0 ± 12.0/ 107.0 ± 11.7	72.5 ± 8.5/ 73.2 ± 8.2	Low PAL: 75.2%/64.3%	Current: 32.5%/13.4%	No info	No info
Rebholz et al., 2016 [48]	DM (9.2%/13.0%) Obesity	35.9%/32.7%	No info	122.3 ± 19.1/ 119.6 ± 18.3	No info	PAI: 2.3 ± 0.7/ 2.6 ± 0.8	Current: 35.7%/17.2%	No info	No info
Santiago-Torres et al., 2020 [49]	MetS (42.0%/25.0%)	No info	No info	120.0 ± 10.5/ 121.0 ± 13.4	70.6 ± 6.9/ 71.0 ± 8.0	No info	No info	No info	No info
Tangney et al., 2015 [50]	Hypertension	Fung DASH Score: 23.0%/36.0%	Toledo DASH Score: 29.5 ± 4.4/30.7 ± 2.5 Fung DASH Score: 29.9 ± 5.7/31.0 ± 5.4 Folsom DASH Score: 30.3 ± 3.8/29.6 ± 4.5	Fung DASH Score: 128.0 ± 18.0/132.0 ± 20.0	Fung DASH Score: 70.0 ± 11.0/ 69.0 ± 12.0	No info	No info	No info	No info

Refers to low- and high-adherence DASH diet group. * Expressed as median (25th and 75th percentiles). Abbreviations: BMI: body mass index; BP: blood pressure; CHD: coronary heart disease; CKD: chronic kidney disease; CVD: cardio-vascular disease; DASH: dietary approaches to stop hypertension; DBP: diastolic blood pressure; DM: diabetes mellitus; GL: glucose; HDL: high-density lipoprotein; HTN: hypertension; MET: Metabolic Equivalent Task; MetS: metabolic syndrome; PA: physical activity; PAI: physical activity index; PAQ-C: physical activity questionnaire for older children; SBP: systolic blood pressure; TG: triglycerides. ^1^ Generated using the intensity and time spent performing each type of activity, assessed using a physical activity questionnaire. ^2^ Assessed using a modified Paffenbarger questionnaire from the Harvard Alumni Activity Survey; the score was defined by multiplying the self-reported hours spent doing heavy, moderate, light or sedentary activities over 24 h by weighing factors that paralleled the rate of oxygen consumption of each activity.

## Supplementary Materials

The following supporting information can be downloaded at https://www.mdpi.com/article/10.3390/metabo13080924/s1: Supplementary Figure S1. Subgroup analysis for SBP according to the use of antihypertensive medication. Figure S2. Subgroup analysis for SBP according to the use of antihypertensive medication; Figure S3. Subgroup analysis for SBP based on the study design; Figure S4. Subgroup analysis for DBP based on the study design; Figure S5. Subgroup analysis for SBP based on the different continent; Figure S6. Subgroup analysis for DBP based on the different continents; Figure S7. Leave-one-out analysis for the SBP outcome; Figure S8. Leave-one-out analysis for the DBP outcome; Figure S9. Funnel plot for the SBP outcome; Figure S10. Funnel plot for the DBP outcome; Table S1. PRISMA 2020 checklist; Table S2. MOOSE checklist; Table S3. Search strategy for identifying observational studies on Pubmed; Table S4. Quality appraisal of cohort studies using the JBI Tool; Table S5. Quality appraisal of cross-sectional studies using the JBI Tool; Table S6. Quality appraisal of case-control studies using the JBI Tool; Table S7. Quality appraisal of randomized control trials using the RoB 2.0 Tool; Table S8. Quality appraisal of randomized control trials using the CASP.
