# Supplementary material for: Impact of the Level of Adherence to the DASH Diet on Blood Pressure: A Systematic Review and Meta-Analysis
Source: Metabolites. 2023 Aug 7;13(8):924. doi: 10.3390/metabo13080924 (PMC10456469; doi:10.3390/metabo13080924)

Contents

Supplementary Table S1. PRISMA 2020 checklist

Supplementary Table S2. MOOSE checklist

Supplementary Table S3. Search strategy for identifying observational studies on Pubmed

Supplementary Table S4. Quality appraisal of cohort studies using the JBI Tool

Supplementary Table S5. Quality appraisal of cross-sectional studies using the JBI Tool

Supplementary Table S6. Quality appraisal of case-control studies using the JBI Tool

Supplementary Table S7. Quality appraisal of randomized control trials using the RoB 2.0 Tool

Supplementary Table S8. Quality appraisal of randomized control trials using the CASP

Supplementary Figure S1. Subgroup analysis for SBP according to the use of antihypertensive medication

Supplementary Figure S2. Subgroup analysis for SBP according to the use of antihypertensive medication

Supplementary Figure S3. Subgroup analysis for SBP based on the study design

Supplementary Figure S4. Subgroup analysis for DBP based on the study design

Supplementary Figure S5. Subgroup analysis for SBP based on the different continents

Supplementary Figure S6. Subgroup analysis for DBP based on the different continents

Supplementary Figure S7. Leave-one-out analysis for the SBP outcome

Supplementary Figure S8. Leave-one-out analysis for the DBP outcome

Supplementary Figure S9. Funnel plot for the SBP outcome

Supplementary Figure S10. Funnel plot for the DBP outcome

Supplementary Table S1. PRISMA 2020 checklist

Section and Topic	Item #	Checklist item	Location where item is reported
**TITLE**			
Title	1	Identify the report as a systematic review.	Page 1
**ABSTRACT**			
Abstract	2	See the PRISMA 2020 for Abstracts checklist.	Page 1
**INTRODUCTION**			
Rationale	3	Describe the rationale for the review in the context of existing knowledge.	Pages 1-2
Objectives	4	Provide an explicit statement of the objective(s) or question(s) the review addresses.	Page 2
**METHODS**			
Eligibility criteria	5	Specify the inclusion and exclusion criteria for the review and how studies were grouped for the syntheses.	Page 2
Information sources	6	Specify all databases, registers, websites, organisations, reference lists and other sources searched or consulted to identify studies. Specify the date when each source was last searched or consulted.	Page 2
Search strategy	7	Present the full search strategies for all databases, registers and websites, including any filters and limits used.	Supplementary Table 3
Selection process	8	Specify the methods used to decide whether a study met the inclusion criteria of the review, including how many reviewers screened each record and each report retrieved, whether they worked independently, and if applicable, details of automation tools used in the process.	Page 2
Data collection process	9	Specify the methods used to collect data from reports, including how many reviewers collected data from each report, whether they worked independently, any processes for obtaining or confirming data from study investigators, and if applicable, details of automation tools used in the process.	Page 2
Data items	10a	List and define all outcomes for which data were sought. Specify whether all results that were compatible with each outcome domain in each study were sought (e.g. for all measures, time points, analyses), and if not, the methods used to decide which results to collect.	Page 3
	10b	List and define all other variables for which data were sought (e.g. participant and intervention characteristics, funding sources). Describe any assumptions made about any missing or unclear information.	Page 3
Study risk of bias assessment	11	Specify the methods used to assess risk of bias in the included studies, including details of the tool(s) used, how many reviewers assessed each study and whether they worked independently, and if applicable, details of automation tools used in the process.	Page 3
Effect measures	12	Specify for each outcome the effect measure(s) (e.g. risk ratio, mean difference) used in the synthesis or presentation of results.	Page 3
Synthesis methods	13a	Describe the processes used to decide which studies were eligible for each synthesis (e.g. tabulating the study intervention characteristics and comparing against the planned groups for each synthesis (item #5)).	Page 3
	13b	Describe any methods required to prepare the data for presentation or synthesis, such as handling of missing summary statistics, or data	Page 3

Section and Topic	Item #	Checklist item	Location where item is reported
		conversions.	
	13c	Describe any methods used to tabulate or visually display results of individual studies and syntheses.	Page 3
	13d	Describe any methods used to synthesize results and provide a rationale for the choice(s). If meta-analysis was performed, describe the model(s), method(s) to identify the presence and extent of statistical heterogeneity, and software package(s) used.	Page 3
	13e	Describe any methods used to explore possible causes of heterogeneity among study results (e.g. subgroup analysis, meta-regression).	Page 3
	13f	Describe any sensitivity analyses conducted to assess robustness of the synthesized results.	Page 3
Reporting bias assessment	14	Describe any methods used to assess risk of bias due to missing results in a synthesis (arising from reporting biases).	NA
Certainty assessment	15	Describe any methods used to assess certainty (or confidence) in the body of evidence for an outcome.	Page 3
**RESULTS**			
Study selection	16a	Describe the results of the search and selection process, from the number of records identified in the search to the number of studies included in the review, ideally using a flow diagram.	Figure 1<br>Page 4
	16b	Cite studies that might appear to meet the inclusion criteria, but which were excluded, and explain why they were excluded.	Figure 1
Study characteristics	17	Cite each included study and present its characteristics.	Tables 1-2<br>Pages 5-11
Risk of bias in studies	18	Present assessments of risk of bias for each included study.	Supplementary Tables S4-S8<br>Page 25
Results of individual studies	19	For all outcomes, present, for each study: (a) summary statistics for each group (where appropriate) and (b) an effect estimate and its precision (e.g. confidence/credible interval), ideally using structured tables or plots.	Figures 2-3<br>Pages 23-24
Results of syntheses	20a	For each synthesis, briefly summarise the characteristics and risk of bias among contributing studies.	Pages 23-25
	20b	Present results of all statistical syntheses conducted. If meta-analysis was done, present for each the summary estimate and its precision (e.g. confidence/credible interval) and measures of statistical heterogeneity. If comparing groups, describe the direction of the effect.	Pages 23-24
	20c	Present results of all investigations of possible causes of heterogeneity among study results.	Pages 24-25<br>Supplementary Figures S1-S8
	20d	Present results of all sensitivity analyses conducted to assess the robustness of the synthesized results.	Page 25<br>Supplementary Figures S7-S8

Section and Topic	Item #	Checklist item	Location where item is reported
Reporting biases	21	Present assessments of risk of bias due to missing results (arising from reporting biases) for each synthesis assessed.	NA
Certainty of evidence	22	Present assessments of certainty (or confidence) in the body of evidence for each outcome assessed.	Page 25
**DISCUSSION**			
Discussion	23a	Provide a general interpretation of the results in the context of other evidence.	Page 26
	23b	Discuss any limitations of the evidence included in the review.	Page 27
	23c	Discuss any limitations of the review processes used.	Page 27
	23d	Discuss implications of the results for practice, policy, and future research.	Page 27
**OTHER INFORMATION**			
Registration and protocol	24a	Provide registration information for the review, including register name and registration number, or state that the review was not registered.	Page 2
	24b	Indicate where the review protocol can be accessed, or state that a protocol was not prepared.	Page 2
	24c	Describe and explain any amendments to information provided at registration or in the protocol.	NA
Support	25	Describe sources of financial or non-financial support for the review, and the role of the funders or sponsors in the review.	Page 27
Competing interests	26	Declare any competing interests of review authors.	Page 27
Availability of data, code and other materials	27	Report which of the following are publicly available and where they can be found: template data collection forms; data extracted from included studies; data used for all analyses; analytic code; any other materials used in the review.	Page 27

Supplementary Table S2. MOOSE checklist

Item No	Recommendation	Reported on Page No
Reporting of background should include		
1	Problem definition	1-2
2	Hypothesis statement	2
3	Description of study outcome(s)	2-3
4	Type of exposure or intervention used	2
5	Type of study designs used	2
6	Study population	2
Reporting of search strategy should include		
7	Qualifications of searchers (eg, librarians and investigators)	NA
8	Search strategy, including time period included in the synthesis and key words	2
9	Effort to include all available studies, including contact with authors	2
10	Databases and registries searched	2
11	Search software used, name and version, including special features used (eg, explosion)	NA
12	Use of hand searching (eg, reference lists of obtained articles)	2
13	List of citations located and those excluded, including justification	5, Table 1
14	Method of addressing articles published in languages other than English	2
15	Method of handling abstracts and unpublished studies	2
16	Description of any contact with authors	NA
Reporting of methods should include		
17	Description of relevance or appropriateness of studies assembled for assessing the hypothesis to be tested	2
18	Rationale for the selection and coding of data (eg, sound clinical principles or convenience)	2
19	Documentation of how data were classified and coded (eg, multiple raters, blinding and interrater reliability)	3
20	Assessment of confounding (eg, comparability of cases and controls in studies where appropriate)	NA
21	Assessment of study quality, including blinding of quality assessors, stratification or regression on possible predictors of study results	3

22	Assessment of heterogeneity	3
23	Description of statistical methods (eg, complete description of fixed or random effects models, justification of whether the chosen models account for predictors of study results, dose-response models, or cumulative meta-analysis) in sufficient detail to be replicated	3
24	Provision of appropriate tables and graphics	Tables 1-2<br>Figures 1-3
Reporting of results should include		
25	Graphic summarizing individual study estimates and overall estimate	Figures 2-3
26	Table giving descriptive information for each study included	Tables 1-2
27	Results of sensitivity testing (eg, subgroup analysis)	24-25
28	Indication of statistical uncertainty of findings	23-25
Reporting of discussion should include		
29	Quantitative assessment of bias (eg, publication bias)	25
30	Justification for exclusion (eg, exclusion of non-English language citations)	NA
31	Assessment of quality of included studies	25
Reporting of conclusions should include		
32	Consideration of alternative explanations for observed results	NA
33	Generalization of the conclusions (ie, appropriate for the data presented and within the domain of the literature review)	26-27
34	Guidelines for future research	26-27
35	Disclosure of funding source	

Table S3. Search strategy for identifying observational studies on Pubmed.

Search	Query
#1	DASH diet [MeSH Terms]
#2	DASH diet [Title/Abstract]
#3	DASH [Title/Abstract]
#4	Dietary score [Title/Abstract]
#5	Dietary adherence [Title/Abstract]
#6	1-5/OR
#7	Blood pressure [Title/Abstract]
#8	Blood pressure [MeSH Terms]
#9	Hypertension [MeSH Terms]
#10	Hypertension [MeSH Tems]
#11	7-10/OR
#12	#6 AND #11
The last search performed in November 2022	

Table S4. Quality appraisal of cohort studies using the JBI Tool

**Study ID**	**Q1**	**Q2**	**Q3**	**Q4**	**Q5**	**Q6**	**Q7**	**Q8**	**Q9**	**Q10**	**Q11**
Banerjee et al., 2019	U	Y	Y	Y	U	N	Y	Y	Y	U	Y
Bonaccio et al., 2021	Y	Y	Y	Y	U	U	Y	Y	N	N	Y
Critselis et al., 2020	Y	Y	Y	Y	Y	N	Y	Y	Y	U	Y
Dai et al., 2022	Y	Y	Y	Y	Y	N	Y	U	N	N	Y
Daniel et al., 2021	Y	Y	Y	Y	Y	Y	Y	U	U	U	Y
Francisco et al., 2020	Y	Y	Y	Y	N	U	Y	U	Y	N	Y
Gao et al., 2020	Y	Y	N	Y	N	U	Y	U	N	N	Y
Goyal et al., 2021	Y	Y	N	Y	Y	N	Y	Y	N	N	Y
Hu et al., 2021	Y	Y	Y	Y	Y	N	Y	U	N	N	Y
Jones et al., 2010	Y	Y	Y	Y	Y	U	Y	Y	N	N	Y
Kim et al., 2022	Y	Y	Y	Y	Y	U	Y	Y	N	N	Y
Lin et al., 2011	Y	Y	Y	Y	Y	U	Y	N	N	N	Y
Liu et al., 2017	Y	Y	Y	Y	Y	N	Y	N	Y	N	Y
Mattei et al., 2017	Y	Y	Y	Y	Y	U	Y	N	U	N	Y
Mertens et al., 2018	Y	Y	U	Y	Y	N	Y	Y	N	N	Y
Missikpode et al., 2021	Y	Y	U	Y	Y	U	Y	Y	N	N	Y
Ramezankhani et al., 2021	Y	Y	U	Y	Y	U	Y	Y	N	N	Y
Rebholz et al., 2016	Y	Y	U	Y	Y	U	Y	Y	N	N	Y
Santiago-Torres et al., 2020	Y	Y	U	Y	Y	U	Y	N	N	N	Y
N: No; U: Unclear; Y: Yes											

Table S5. Quality appraisal of cross-sectional studies using the JBI Tool

Study ID	Q1	Q2	Q3	Q4	Q5	Q6	Q7	Q8
Bendinelli et al., 2018	Y	Y	Y	Y	U	U	Y	Y
Chan et al., 2022	Y	Y	Y	Y	Y	N	Y	Y
Ghorabi et al., 2019	Y	Y	N	U	Y	Y	N	Y
Harrington et al., 2013	Y	Y	Y	Y	Y	Y	Y	Y
Ishikawa et al., 2022	Y	Y	Y	Y	Y	Y	Y	Y
Jalilpiran et al., 2020	Y	Y	U	U	Y	U	Y	Y
Kang et al., 2018	Y	Y	U	Y	Y	Y	U	Y
Khodarahmi et al., 2021	Y	Y	Y	U	Y	Y	Y	Y
Köroğlu & Adıgüzel. 2020	Y	Y	N	N	N	N	Y	Y
Mackenbach et al., 2018	Y	Y	Y	Y	Y	Y	Y	Y
Mousavi et al., 2020	Y	Y	N	Y	Y	Y	U	Y
Navarro-Prado et al., 2020	N	Y	Y	Y	Y	Y	Y	Y
Nilsson et al., 2019	Y	Y	Y	U	Y	U	Y	Y
Tangney et al., 2015	Y	Y	Y	U	Y	Y	Y	Y
N: No; U: Unclear; Y: Yes								

Table S6. Quality appraisal of case-control studies using the JBI Tool

**Study ID**	**Q1**	**Q2**	**Q3**	**Q4**	**Q5**	**Q6**	**Q7**	**Q8**	**Q9**	**Q10**
Jayedi et al., 2019	Y	Y	Y	Y	Y	Y	Y	Y	Y	Y
Y: Yes										

Table S7. Quality appraisal of randomized control trials using the RoB 2.0 Tool

**Study ID**	**Domain 1**	**Domain 2**	**Domain 3**	**Domain 4**	**Domain 5**	**Overall**
Epstein et al., 2012	Low	Some concerns	Low	Some concerns	Some concerns	Some concerns
Gao et al., 2021	Low	Low	Some concerns	Low	Some concerns	Some concerns

Table S8. Quality appraisal of randomized control trials using the CASP

Study ID	Domain 1	Domain 2	Domain 3	Domain 4	Domain 5	Domain 6	Domain 7	Domain 8	Domain 9	Domain 10	Domain 11
Glenn et al., 2021	Yes	Yes	Yes	No	Yes	Can’t Tell	Yes	Yes	Yes	Yes	Can’t Tell

Supplementary Figure S1. Subgroup analysis for SBP according to the use of antihypertensive medication

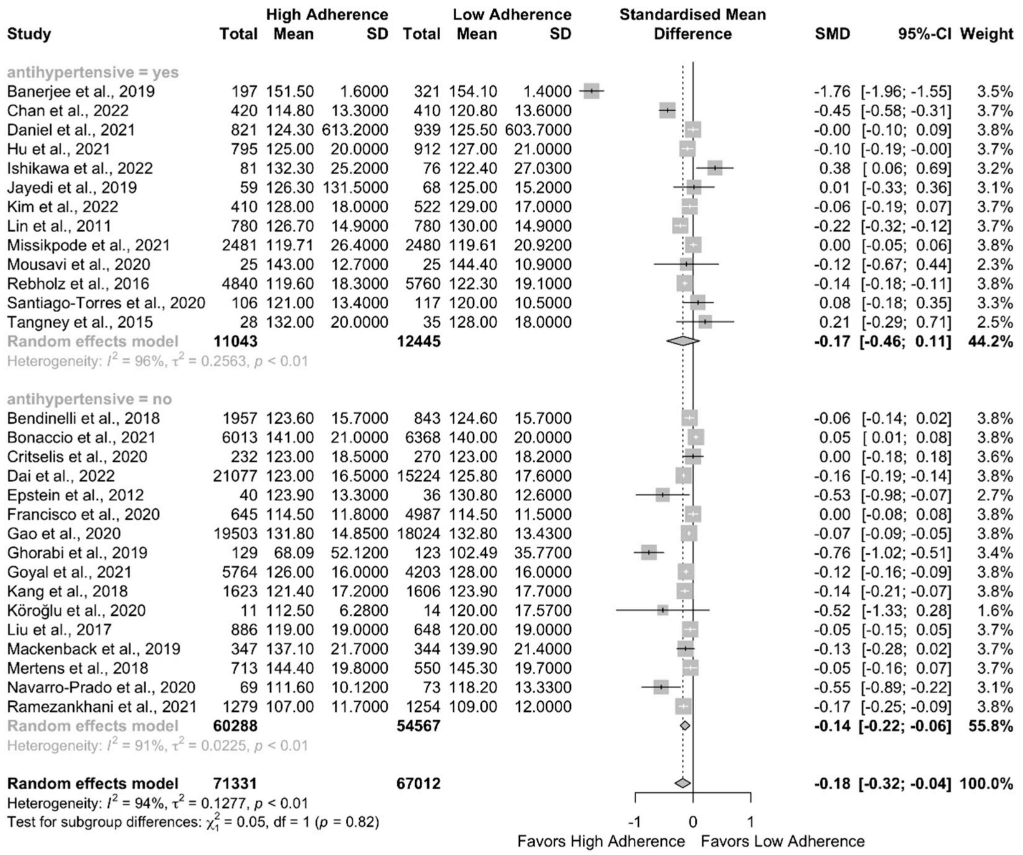

Supplementary Figure S2. Subgroup analysis for DBP according to the use of antihypertensive medication

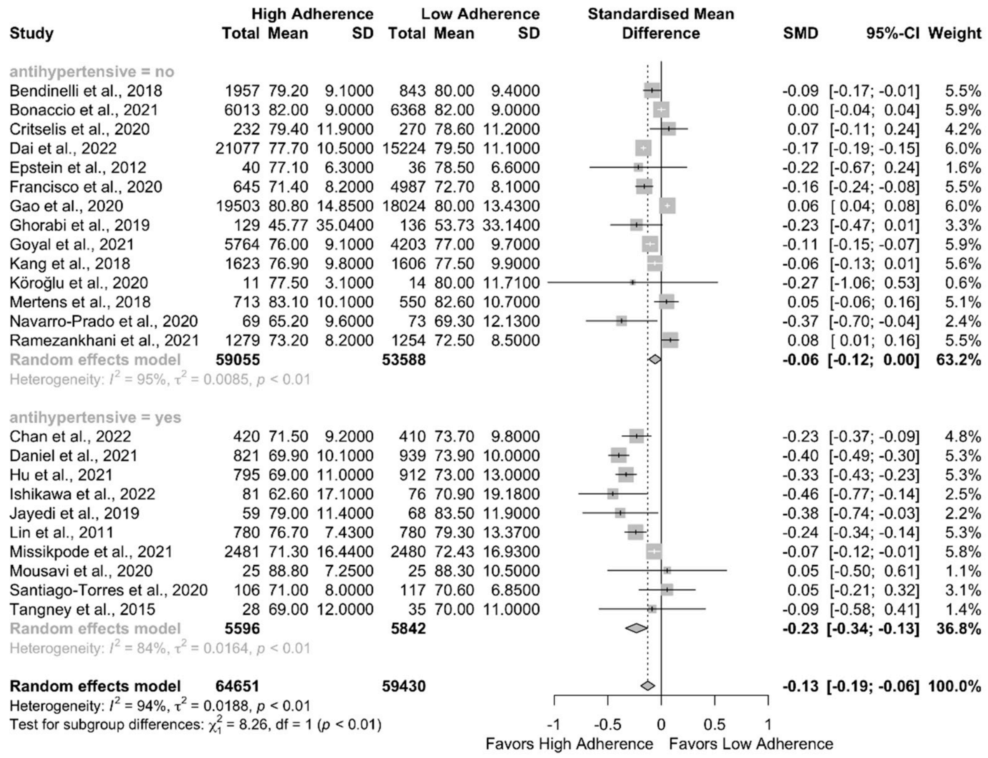

Supplementary Figure S3. Subgroup analysis for SBP based on the study design

Study	High Adherence Total	High Adherence Mean	High Adherence SD	Low Adherence Total	Low Adherence Mean	Low Adherence SD	SMD	95%-CI	Weight
**Study_Design = Prospective observational study**									
Banerjee et al., 2019	197	151.50	1.6000	321	154.10	1.4000	-1.76	[-1.96; -1.55]	3.5%
Bonaccio et al., 2021	6013	141.00	21.0000	6368	140.00	20.0000	0.05	[0.01; 0.08]	3.8%
Critselis et al., 2020	232	123.00	18.5000	270	123.00	18.2000	0.00	[-0.18; 0.18]	3.6%
Dai et al., 2022	21077	123.00	16.5000	15224	125.80	17.6000	-0.16	[-0.19; -0.14]	3.8%
Daniel et al., 2021	821	124.30	613.2000	939	125.50	603.7000	-0.00	[-0.10; 0.09]	3.8%
Francisco et al., 2020	645	114.50	11.8000	4987	114.50	11.5000	0.00	[-0.08; 0.08]	3.8%
Gao et al., 2020	19503	131.80	14.8500	18024	132.80	13.4300	-0.07	[-0.09; -0.05]	3.8%
Goyal et al., 2021	5764	126.00	16.0000	4203	128.00	16.0000	-0.12	[-0.16; -0.09]	3.8%
Hu et al., 2021	795	125.00	20.0000	912	127.00	21.0000	-0.10	[-0.19; -0.00]	3.7%
Kim et al., 2022	410	128.00	18.0000	522	129.00	17.0000	-0.06	[-0.19; 0.07]	3.7%
Lin et al., 2011	780	126.70	14.9000	780	130.00	14.9000	-0.22	[-0.32; -0.12]	3.7%
Liu et al., 2017	886	119.00	19.0000	648	120.00	19.0000	-0.05	[-0.15; 0.05]	3.7%
Mertens et al., 2018	713	144.40	19.8000	550	145.30	19.7000	-0.05	[-0.16; 0.07]	3.7%
Missikpode et al., 2021	2481	119.71	26.4000	2480	119.61	20.9200	0.00	[-0.05; 0.06]	3.8%
Ramezankhani et al., 2021	1279	107.00	11.7000	1254	109.00	12.0000	-0.17	[-0.25; -0.09]	3.8%
Rebholz et al., 2016	4840	119.60	18.3000	5760	122.30	19.1000	-0.14	[-0.18; -0.11]	3.8%
Santiago-Torres et al., 2020	106	121.00	13.4000	117	120.00	10.5000	0.08	[-0.18; 0.35]	3.3%
**Random effects model**	**66542**			**63359**			**-0.16**	**[-0.35; 0.03]**	**63.2%**
Heterogeneity: $I^2 = 96\%$, $\tau^2 = 0.1570$, $p < 0.01$									
**Study_Design = Cross-sectional observational study**									
Bendinelli et al., 2018	1957	123.60	15.7000	843	124.60	15.7000	-0.06	[-0.14; 0.02]	3.8%
Chan et al., 2022	420	114.80	13.3000	410	120.80	13.6000	-0.45	[-0.58; -0.31]	3.7%
Ghorabi et al., 2019	129	68.09	52.1200	123	102.49	35.7700	-0.76	[-1.02; -0.51]	3.4%
Ishikawa et al., 2022	81	132.30	25.2000	76	122.40	27.0300	0.38	[0.06; 0.69]	3.2%
Jayedi et al., 2019	59	126.30	131.5000	68	125.00	15.2000	0.01	[-0.33; 0.36]	3.1%
Kang et al., 2018	1623	121.40	17.2000	1606	123.90	17.7000	-0.14	[-0.21; -0.07]	3.8%
Köroğlu et al., 2020	11	112.50	6.2800	14	120.00	17.5700	-0.52	[-1.33; 0.28]	1.6%
Mackenback et al., 2019	347	137.10	21.7000	344	139.90	21.4000	-0.13	[-0.28; 0.02]	3.7%
Mousavi et al., 2020	25	143.00	12.7000	25	144.40	10.9000	-0.12	[-0.67; 0.44]	2.3%
Navarro-Prado et al., 2020	69	111.60	10.1200	73	118.20	13.3300	-0.55	[-0.89; -0.22]	3.1%
Tangney et al., 2015	28	132.00	20.0000	35	128.00	18.0000	0.21	[-0.29; 0.71]	2.5%
**Random effects model**	**4749**			**3617**			**-0.19**	**[-0.39; 0.01]**	**34.1%**
Heterogeneity: $I^2 = 84\%$, $\tau^2 = 0.0879$, $p < 0.01$									
**Study_Design = Randomized controlled trial**									
Epstein et al., 2012	40	123.90	13.3000	36	130.80	12.6000	-0.53	[-0.98; -0.07]	2.7%
**Random effects model**	**71331**			**67012**			**-0.18**	**[-0.32; -0.04]**	**100.0%**
Heterogeneity: $I^2 = 94\%$, $\tau^2 = 0.1277$, $p < 0.01$									
Test for subgroup differences: $\chi^2_2 = 2.12$, df = 2 ($p = 0.35$)									

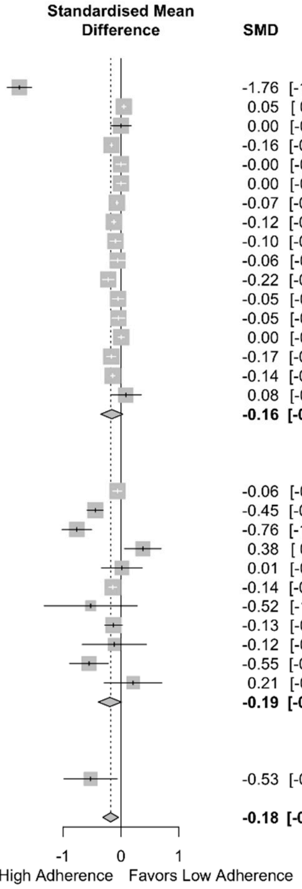

Supplementary Figure S4. Subgroup analysis for DBP based on the study design

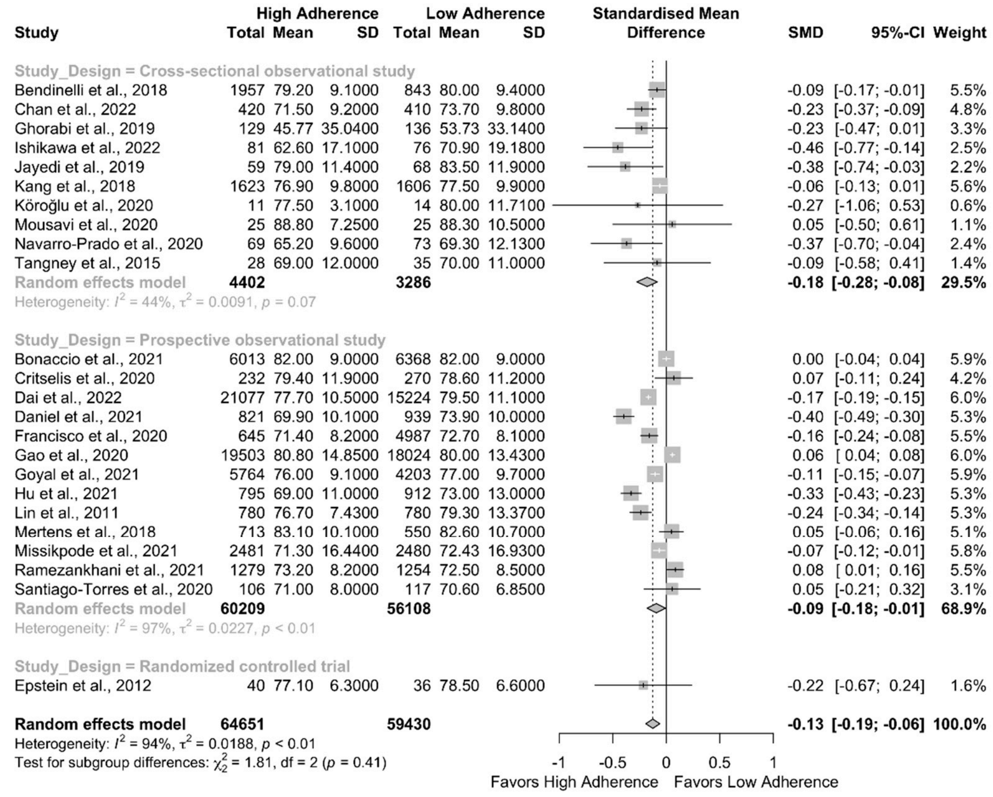

Supplementary Figure S5. Subgroup analysis for SBP based on the different continents

Study	High Adherence Total	High Adherence Mean	High Adherence SD	Low Adherence Total	Low Adherence Mean	Low Adherence SD	SMD	95%-CI	Weight
**Continent = North America**									
Banerjee et al., 2019	197	151.50	1.6000	321	154.10	1.4000	-1.76	[-1.96; -1.55]	3.5%
Daniel et al., 2021	821	124.30	613.2000	939	125.50	603.7000	-0.00	[-0.10; 0.09]	3.8%
Epstein et al., 2012	40	123.90	13.3000	36	130.80	12.6000	-0.53	[-0.98; -0.07]	2.7%
Goyal et al., 2021	5764	126.00	16.0000	4203	128.00	16.0000	-0.12	[-0.16; -0.09]	3.8%
Hu et al., 2021	795	125.00	20.0000	912	127.00	21.0000	-0.10	[-0.19; -0.00]	3.7%
Ishikawa et al., 2022	81	132.30	25.2000	76	122.40	27.0300	0.38	[0.06; 0.69]	3.2%
Kim et al., 2022	410	128.00	18.0000	522	129.00	17.0000	-0.06	[-0.19; 0.07]	3.7%
Lin et al., 2011	780	126.70	14.9000	780	130.00	14.9000	-0.22	[-0.32; -0.12]	3.7%
Liu et al., 2017	886	119.00	19.0000	648	120.00	19.0000	-0.05	[-0.15; 0.05]	3.7%
Missikpode et al., 2021	2481	119.71	26.4000	2480	119.61	20.9200	0.00	[-0.05; 0.06]	3.8%
Rebholz et al., 2016	4840	119.60	18.3000	5760	122.30	19.1000	-0.14	[-0.18; -0.11]	3.8%
Santiago-Torres et al., 2020	106	121.00	13.4000	117	120.00	10.5000	0.08	[-0.18; 0.35]	3.3%
Tangney et al., 2015	28	132.00	20.0000	35	128.00	18.0000	0.21	[-0.29; 0.71]	2.5%
**Random effects model**	**17229**			**16829**			**-0.18**	**[-0.46; 0.10]**	**45.3%**
Heterogeneity: $I^2 = 96\%$, $\tau^2 = 0.2523$, $p < 0.01$									
**Continent = Europe**									
Bendinelli et al., 2018	1957	123.60	15.7000	843	124.60	15.7000	-0.06	[-0.14; 0.02]	3.8%
Bonaccio et al., 2021	6013	141.00	21.0000	6368	140.00	20.0000	0.05	[0.01; 0.08]	3.8%
Critselis et al., 2020	232	123.00	18.5000	270	123.00	18.2000	0.00	[-0.18; 0.18]	3.6%
Mackenback et al., 2019	347	137.10	21.7000	344	139.90	21.4000	-0.13	[-0.28; 0.02]	3.7%
Mertens et al., 2018	713	144.40	19.8000	550	145.30	19.7000	-0.05	[-0.16; 0.07]	3.7%
Navarro-Prado et al., 2020	69	111.60	10.1200	73	118.20	13.3300	-0.55	[-0.89; -0.22]	3.1%
**Random effects model**	**9331**			**8448**			**-0.06**	**[-0.15; 0.03]**	**21.6%**
Heterogeneity: $I^2 = 78\%$, $\tau^2 = 0.0084$, $p < 0.01$									
**Continent = Worldwide**									
Chan et al., 2022	420	114.80	13.3000	410	120.80	13.6000	-0.45	[-0.58; -0.31]	3.7%
**Continent = Asia**									
Dai et al., 2022	21077	123.00	16.5000	15224	125.80	17.6000	-0.16	[-0.19; -0.14]	3.8%
Gao et al., 2020	19503	131.80	14.8500	18024	132.80	13.4300	-0.07	[-0.09; -0.05]	3.8%
Ghorabi et al., 2019	129	68.09	52.1200	123	102.49	35.7700	-0.76	[-1.02; -0.51]	3.4%
Jayedi et al., 2019	59	126.30	131.5000	68	125.00	15.2000	0.01	[-0.33; 0.36]	3.1%
Kang et al., 2018	1623	121.40	17.2000	1606	123.90	17.7000	-0.14	[-0.21; -0.07]	3.8%
Köroğlu et al., 2020	11	112.50	6.2800	14	120.00	17.5700	-0.52	[-1.33; 0.28]	1.6%
Mousavi et al., 2020	25	143.00	12.7000	25	144.40	10.9000	-0.12	[-0.67; 0.44]	2.3%
Ramezankhani et al., 2021	1279	107.00	11.7000	1254	109.00	12.0000	-0.17	[-0.25; -0.09]	3.8%
**Random effects model**	**43706**			**36338**			**-0.21**	**[-0.38; -0.04]**	**25.6%**
Heterogeneity: $I^2 = 90\%$, $\tau^2 = 0.0412$, $p < 0.01$									
**Continent = South America**									
Francisco et al., 2020	645	114.50	11.8000	4987	114.50	11.5000	0.00	[-0.08; 0.08]	3.8%
**Random effects model**	**71331**			**67012**			**-0.18**	**[-0.32; -0.04]**	**100.0%**

Heterogeneity: $I^2 = 94\%$, $\tau^2 = 0.1277$, $p < 0.01$

Test for subgroup differences: $\chi^2_4 = 32.44$, df = 4 ($p < 0.01$)

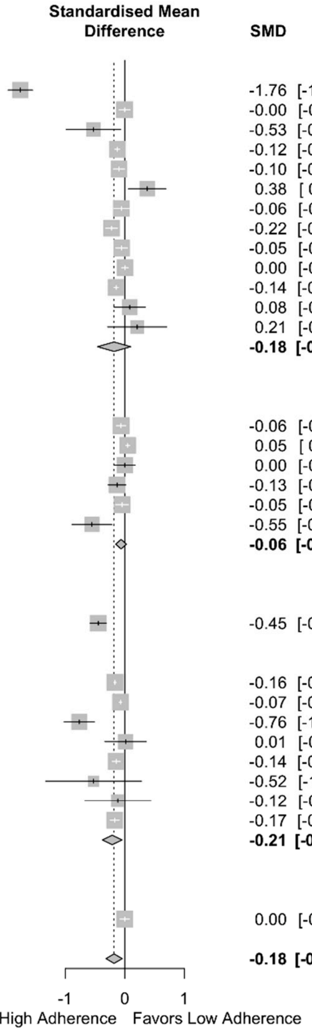

Supplementary Figure S6. Subgroup analysis for DBP based on the different continents

Study	High Adherence Total	High Adherence Mean	High Adherence SD	Low Adherence Total	Low Adherence Mean	Low Adherence SD	SMD	95%-CI	Weight
**Continents = Europe**									
Bendinelli et al., 2018	1957	79.20	9.1000	843	80.00	9.4000	-0.09	[-0.17; -0.01]	5.5%
Bonaccio et al., 2021	6013	82.00	9.0000	6368	82.00	9.0000	0.00	[-0.04; 0.04]	5.9%
Critselis et al., 2020	232	79.40	11.9000	270	78.60	11.2000	0.07	[-0.11; 0.24]	4.2%
Mertens et al., 2018	713	83.10	10.1000	550	82.60	10.7000	0.05	[-0.06; 0.16]	5.1%
Navarro-Prado et al., 2020	69	65.20	9.6000	73	69.30	12.1300	-0.37	[-0.70; -0.04]	2.4%
**Random effects model**	**8984**			**8104**			**-0.02**	**[-0.09; 0.05]**	**23.2%**
Heterogeneity: $I^2 = 61\%$, $\tau^2 = 0.0025$, $p = 0.04$									
**Continents = Worldwide**									
Chan et al., 2022	420	71.50	9.2000	410	73.70	9.8000	-0.23	[-0.37; -0.09]	4.8%
**Continents = Asia**									
Dai et al., 2022	21077	77.70	10.5000	15224	79.50	11.1000	-0.17	[-0.19; -0.15]	6.0%
Gao et al., 2020	19503	80.80	14.8500	18024	80.00	13.4300	0.06	[0.04; 0.08]	6.0%
Ghorabi et al., 2019	129	45.77	35.0400	136	53.73	33.1400	-0.23	[-0.47; 0.01]	3.3%
Jayedi et al., 2019	59	79.00	11.4000	68	83.50	11.9000	-0.38	[-0.74; -0.03]	2.2%
Kang et al., 2018	1623	76.90	9.8000	1606	77.50	9.9000	-0.06	[-0.13; 0.01]	5.6%
Köroğlu et al., 2020	11	77.50	3.1000	14	80.00	11.7100	-0.27	[-1.06; 0.53]	0.6%
Mousavi et al., 2020	25	88.80	7.2500	25	88.30	10.5000	0.05	[-0.50; 0.61]	1.1%
Ramezankhani et al., 2021	1279	73.20	8.2000	1254	72.50	8.5000	0.08	[0.01; 0.16]	5.5%
**Random effects model**	**43706**			**36351**			**-0.07**	**[-0.18; 0.04]**	**30.4%**
Heterogeneity: $I^2 = 97\%$, $\tau^2 = 0.0153$, $p < 0.01$									
**Continents = North America**									
Daniel et al., 2021	821	69.90	10.1000	939	73.90	10.0000	-0.40	[-0.49; -0.30]	5.3%
Epstein et al., 2012	40	77.10	6.3000	36	78.50	6.6000	-0.22	[-0.67; 0.24]	1.6%
Goyal et al., 2021	5764	76.00	9.1000	4203	77.00	9.7000	-0.11	[-0.15; -0.07]	5.9%
Hu et al., 2021	795	69.00	11.0000	912	73.00	13.0000	-0.33	[-0.43; -0.23]	5.3%
Ishikawa et al., 2022	81	62.60	17.1000	76	70.90	19.1800	-0.46	[-0.77; -0.14]	2.5%
Lin et al., 2011	780	76.70	7.4300	780	79.30	13.3700	-0.24	[-0.34; -0.14]	5.3%
Missikpode et al., 2021	2481	71.30	16.4400	2480	72.43	16.9300	-0.07	[-0.12; -0.01]	5.8%
Santiago-Torres et al., 2020	106	71.00	8.0000	117	70.60	6.8500	0.05	[-0.21; 0.32]	3.1%
Tangney et al., 2015	28	69.00	12.0000	35	70.00	11.0000	-0.09	[-0.58; 0.41]	1.4%
**Random effects model**	**10896**			**9578**			**-0.21**	**[-0.32; -0.10]**	**36.1%**
Heterogeneity: $I^2 = 87\%$, $\tau^2 = 0.0189$, $p < 0.01$									
**Continents = South America**									
Francisco et al., 2020	645	71.40	8.2000	4987	72.70	8.1000	-0.16	[-0.24; -0.08]	5.5%
**Random effects model**	**64651**			**59430**			**-0.13**	**[-0.19; -0.06]**	**100.0%**
Heterogeneity: $I^2 = 94\%$, $\tau^2 = 0.0188$, $p < 0.01$									
Test for subgroup differences: $\chi^2_4 = 15.45$, df = 4 ($p < 0.01$)									

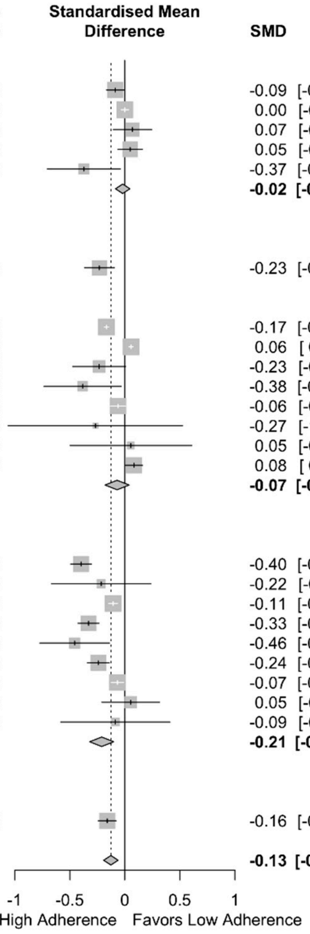

Supplementary Figure S7. Leave-one-out analysis for the SBP outcome

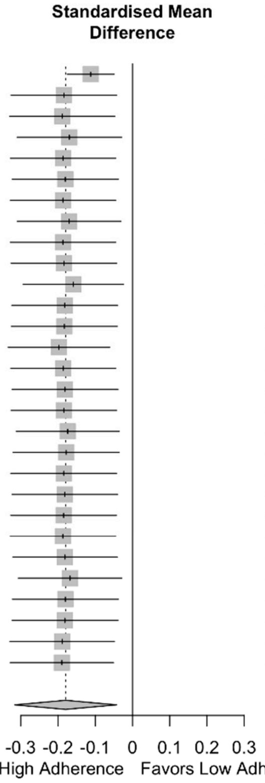

Study	SMD	95%-CI	P-value	Tau2	Tau	I2
Omitting Banerjee et al., 2019	-0.11	[-0.18; -0.05]	< 0.01	0.0213	0.1458	88%
Omitting Bendinelli et al., 2018	-0.18	[-0.33; -0.04]	0.01	0.1330	0.3647	94%
Omitting Bonaccio et al., 2021	-0.19	[-0.33; -0.05]	< 0.01	0.1312	0.3622	93%
Omitting Chan et al., 2022	-0.17	[-0.31; -0.03]	0.02	0.1301	0.3607	94%
Omitting Critselis et al., 2020	-0.19	[-0.33; -0.05]	< 0.01	0.1320	0.3633	94%
Omitting Dai et al., 2022	-0.18	[-0.32; -0.04]	0.01	0.1336	0.3656	94%
Omitting Daniel et al., 2021	-0.19	[-0.33; -0.05]	< 0.01	0.1321	0.3635	94%
Omitting Epstein et al., 2012	-0.17	[-0.31; -0.03]	0.02	0.1290	0.3591	94%
Omitting Francisco et al., 2020	-0.19	[-0.33; -0.05]	< 0.01	0.1321	0.3635	94%
Omitting Gao et al., 2020	-0.18	[-0.33; -0.04]	0.01	0.1331	0.3648	94%
Omitting Ghorabi et al., 2019	-0.16	[-0.29; -0.02]	0.02	0.1198	0.3462	94%
Omitting Goyal et al., 2021	-0.18	[-0.32; -0.04]	0.01	0.1335	0.3654	94%
Omitting Hu et al., 2021	-0.18	[-0.33; -0.04]	0.01	0.1333	0.3650	94%
Omitting Ishikawa et al., 2022	-0.20	[-0.33; -0.06]	< 0.01	0.1226	0.3502	94%
Omitting Jayedi et al., 2019	-0.19	[-0.33; -0.05]	< 0.01	0.1314	0.3625	94%
Omitting Kang et al., 2018	-0.18	[-0.32; -0.04]	0.01	0.1335	0.3654	94%
Omitting Kim et al., 2022	-0.18	[-0.33; -0.04]	0.01	0.1328	0.3645	94%
Omitting Köroğlu et al., 2020	-0.17	[-0.31; -0.04]	0.01	0.1292	0.3594	94%
Omitting Lin et al., 2011	-0.18	[-0.32; -0.04]	0.01	0.1334	0.3653	94%
Omitting Liu et al., 2017	-0.18	[-0.33; -0.04]	0.01	0.1328	0.3645	94%
Omitting Mackenback et al., 2019	-0.18	[-0.32; -0.04]	0.01	0.1333	0.3651	94%
Omitting Mertens et al., 2018	-0.19	[-0.33; -0.04]	0.01	0.1327	0.3643	94%
Omitting Missikpode et al., 2021	-0.19	[-0.33; -0.05]	< 0.01	0.1321	0.3634	94%
Omitting Mousavi et al., 2020	-0.18	[-0.32; -0.04]	0.01	0.1313	0.3624	94%
Omitting Navarro-Prado et al., 2020	-0.17	[-0.31; -0.03]	0.02	0.1280	0.3577	94%
Omitting Ramezankhani et al., 2021	-0.18	[-0.32; -0.04]	0.01	0.1336	0.3655	94%
Omitting Rebholz et al., 2016	-0.18	[-0.32; -0.04]	0.01	0.1336	0.3655	94%
Omitting Santiago-Torres et al., 2020	-0.19	[-0.33; -0.05]	< 0.01	0.1305	0.3612	94%
Omitting Tangney et al., 2015	-0.19	[-0.33; -0.05]	< 0.01	0.1286	0.3586	94%
**Random effects model**	**-0.18**	**[-0.32; -0.04]**	**0.01**	**0.1277**	**0.3573**	**94%**

Supplementary Figure S8. Leave-one-out analysis for the DBP outcome

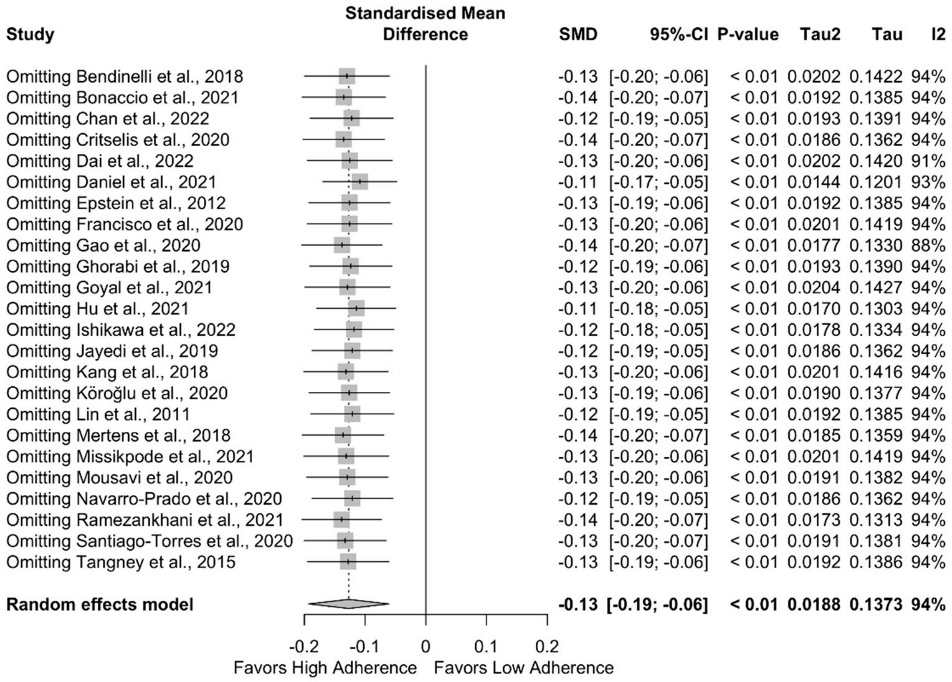

Supplementary Figure S9. Funnel plot for the SBP outcome

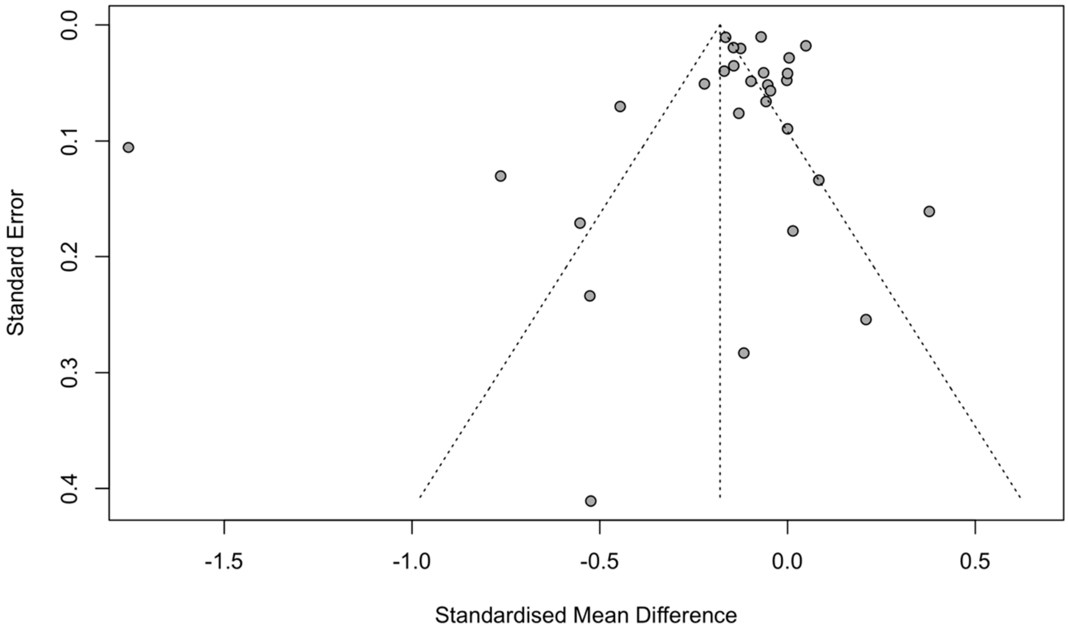

Supplementary Figure S9. Funnel plot for the DBP outcome

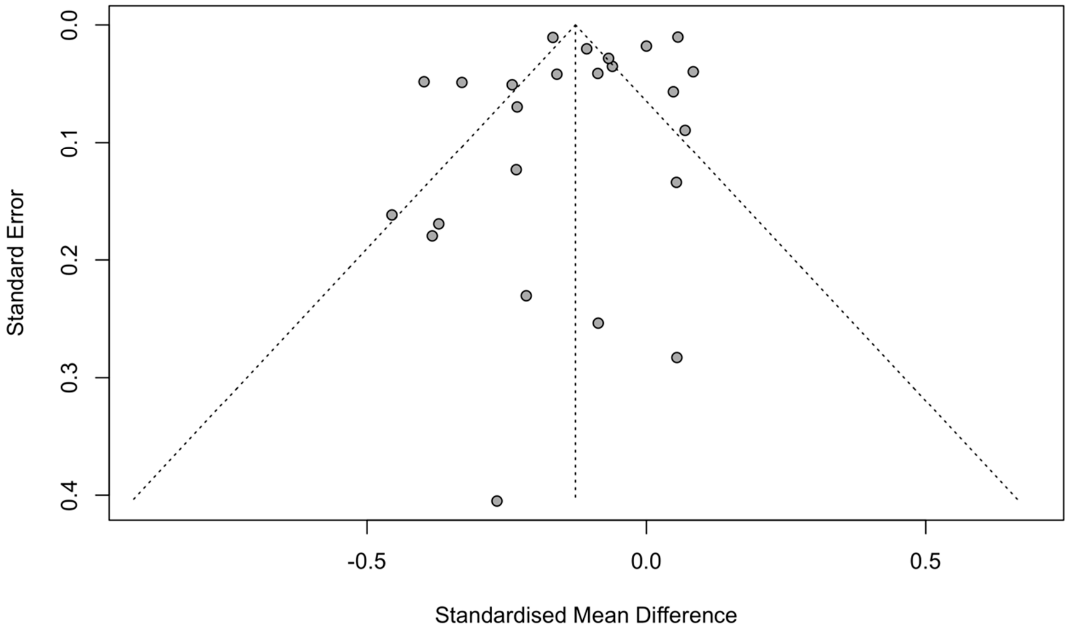

Supplement: Supplementary file 1 [file metabolites-13-00924-s001.zip › metabolites-2486196-supplementary.pdf]
